# Particulate matter impairs immune system function by up-regulating inflammatory pathways and decreasing pathogen response gene expression

**DOI:** 10.1038/s41598-023-39921-w

**Published:** 2023-08-07

**Authors:** Damariz Marín-Palma, Geysson Javier Fernandez, Julian Ruiz-Saenz, Natalia A. Taborda, Maria T. Rugeles, Juan C. Hernandez

**Affiliations:** 1 Infettare, Facultad de Medicina, Universidad Cooperativa de Colombia, Medellín, Colombia; 2 Grupo Inmunovirología, Facultad de Medicina, Universidad de Antioquia UdeA, Medellín, Colombia; 3 Grupo Biología y Control de Enfermedades Infecciosas BCEI, Universidad de Antioquia-UdeA, Medellín, Colombia; 4 Grupo de Investigación en Ciencias Animales GRICA, Universidad Cooperativa de Colombia, Bucaramanga, Colombia; 5 Grupo de Investigaciones Biomédicas Uniremington, Programa de Medicina, Facultad de Ciencias de La Salud, Corporación Universitaria Remington, Medellín, Colombia; 6 Universidad Cooperativa de Colombia, Campus Medellín-Envigado, Medellín, Colombia

**Keywords:** Cytokines, RNA, Environmental impact, Immunology, Transcriptomics

## Abstract

Airborne particulate matter produced by industrial sources and automobiles has been linked to increased susceptibility to infectious diseases and it is known to be recognized by cells of the immune system. The molecular mechanisms and changes in gene expression profiles induced in immune cells by PM have not been fully mapped out or systematically integrated. Here, we use RNA-seq to analyze mRNA profiles of human peripheral blood mononuclear cells after exposure to coarse particulate matter (PM_10_). Our analyses showed that PM_10_ was able to reprogram the expression of 1,196 genes in immune cells, including activation of a proinflammatory state with an increase in cytokines and chemokines. Activation of the IL-36 signaling pathway and upregulation of chemokines involved in neutrophil and monocyte recruitment suggest mechanisms for inflammation upon PM exposure, while NK cell-recruiting chemokines are repressed. PM exposure also increases transcription factors associated with inflammatory pathways (*e.g.,* JUN, RELB, NFKB2, etc.) and reduces expression of RNases and pathogen response genes CAMP, DEFAs, AZU1, APOBEC3A and LYZ. Our analysis across gene regulatory and signaling pathways suggests that PM plays a role in the dysregulation of immune cell functions, relevant for antiviral responses and general host defense against pathogens.

## Introduction

Air pollution continues to be a public health crisis worldwide, with most people living exposed to harmful pollutants and causing over 6.6 million deaths annually. High population density, fuel usage and industry growth are involved in the increased pollution levels with severe consequences for human health^1^. Harmful health effects of air pollutants, such as those caused by particulate matter (PM), have been widely reported. Specifically, lung diseases (i.e., chronic obstructive pulmonary disease -COPD-, asthma, respiratory infectious diseases, and cancer), cardiovascular and neurological disorders and even newborn-related issues have been associated with PM exposure^2–6^. Underlying mechanisms are still unclear but could be related to oxidative stress, tissue damage, genotoxicity, vascular alteration, and airway inflammation induced by these pollutants^7–9^.

The particulate matter PM_10_ (diameter less than 10 µm) is one of the primary pollutants in the air. Its physical–chemical features induce a robust inflammatory response, producing significant harmful effects^7^. Previous studies have demonstrated that PM_10_ can induce inflammation through mechanisms related to inflammasome activation, cell recruitment and increasing production of inflammatory cytokines such as IL-1β, IL-6, I L-8, and TNF-α^10,11^. In addition, in primary human cells, a better model to understand the human response to these air pollutants, we recently described that PM_10_ induces activation of neutrophils, netosis, the release of pro-inflammatory cytokines and neutrophil infiltration of murine lungs^11^. In addition, particulate matter induces hyperreactivity and inflammation through IL-17A and inflammatory γδ T cells. NK cells can activate modulatory mechanisms to control immune effects by expressing Tim-1 and PD-L1 inhibitory molecules, regulating IL-17A production and expansion of γδ T cells. Thus, suggesting a crucial role of NK cells in protecting harmful effects of particulate matter^12^.

This inflammatory response induced by PM_10_ exposure has been associated with exacerbating respiratory diseases, including asthma and COPD^2,13^. Even though the altered immune response also includes decreased antimicrobial responses and loss of tissue homeostasis after PM exposure, increasing the risk of respiratory infections and clinical complications^14,15^. In this regard, PM_10_ might alter pulmonary immune function, increasing pneumonia susceptibility^16^. This alteration in the immune response could be mediated by cell dysfunction or abnormal production of antimicrobial agents, including β-defensin and cathelicidin, increasing the risk of microbial colonization^17^. The antiviral response is also altered by exposition to PM, decreasing type II interferon in people exposed to pollutants, which can increase vulnerability to viral infections^18^.

The transcriptional analysis offers advantages over other methodologies, including an overall gene expression analysis, novel transcripts and non-coding RNAs, instead of a few genes evaluated by traditional RNA quantification strategies^19–21^. Previous studies evaluated the effect of PM_2.5_ on gene expression in human bronchial epithelial and cardiomyocyte using RNA-seq^22,23^. However, little has been explored in gene expression programs modulated by PM10 in immune cells, since systemic effects have been mainly attributed to PM2.5 due to its ability to penetrate to the alveoli, and even pass into the circulation^24^. Nevertheless, different studies have shown that PM10 can also induce extrapulmonary effects, which are mediated by the recruitment of leukocytes^25–27^. Therefore, obtaining mRNA profiles could offer information on gene expression programs modulated by PM10 in immune cells, managing to identify genes and signaling pathways involved that can be used as an intervention target to counteract the harmful effect of PM. For example, the use of RNA-seq in the identification of dysregulated molecular pathways in ovarian cancer has been proposed as an effective strategy to establish early markers for detection, as well as for drug development^28^. As this technology seems helpful in identifying molecular mechanisms involved in the biological effects of a particular matter, we aimed to determine the transcriptional changes upon PM_10_ treatment of primary human immune cells by RNAseq analysis. An in-depth transcriptome analysis allowed us to discover impaired biological processes, such as increased inflammation, including IL-36 signaling and reduced host defense against viruses. Our results show that PM_10_ exposure has an immunosuppressive effect, possibly associated with a higher risk to infectious diseases.

## Results

### PM_10_ induces a differentially expressed mRNA signature in peripheral blood mononuclear cells (PBMCs)

**Figure 1:**
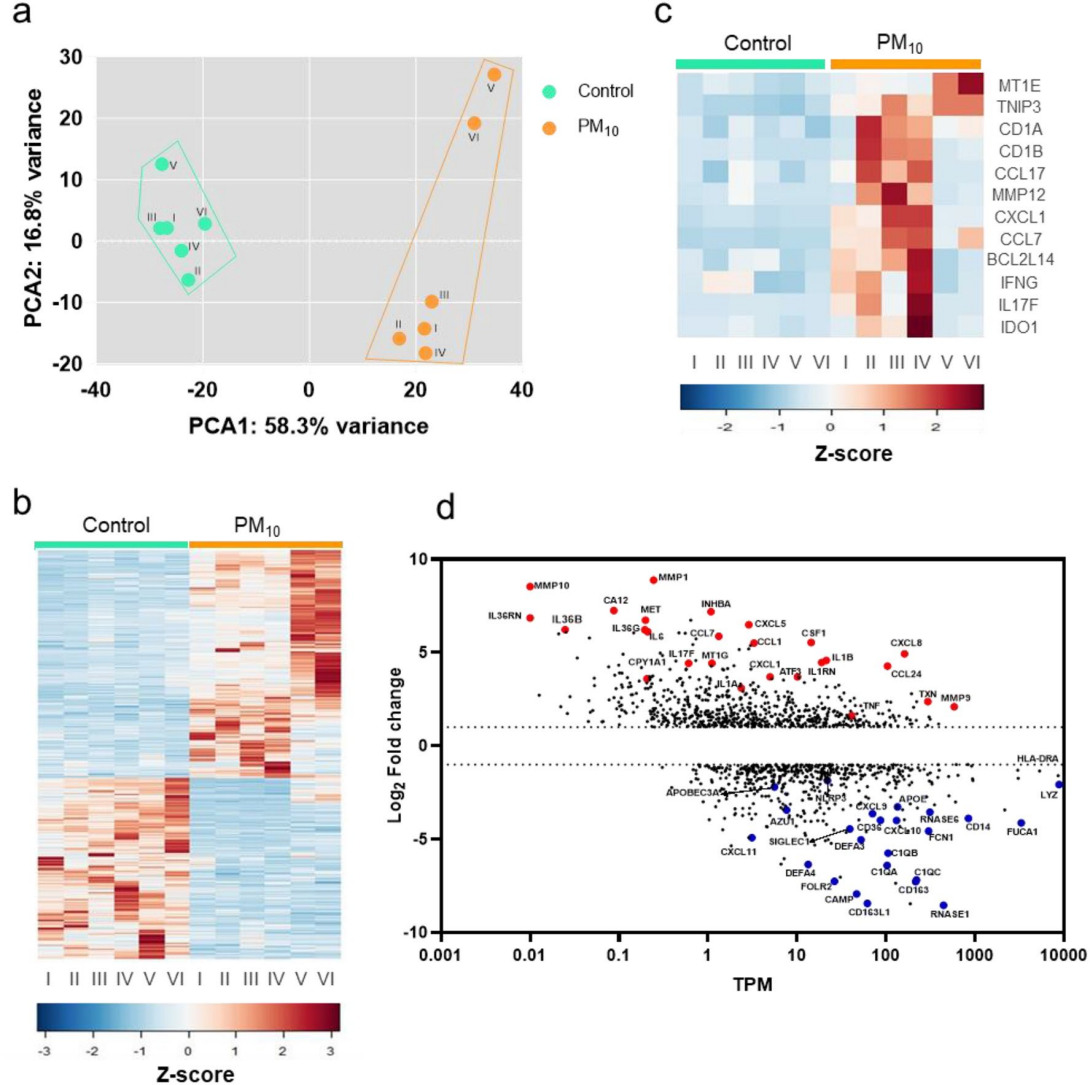
PM_10_ induces a differentially expressed mRNA signature in PBMCs. (**a**) Principal component analysis of gene expression from PBMCs exposed to PM. The percentage of the variance of each principal component (PC1 and PC2) for control (I–VI) and PM exposed cells (I–VI). (**b**) Heatmap of 1196 Z-score normalized differentially expressed genes (DEG) of control and PM-exposed cells. (**c**) Heatmap of 12 Z-score normalized differentially expressed genes with the highest coefficient of variation of control and PM-exposed cells. (**d**) Scatterplot comparing the abundance of transcripts (TPM x-axis) and expression (log_2_-fold change, y-axis). Red and blue dots represent up- and down-regulated genes, respectively (Fold-change > 2 and FDR < 0.05; Wald test).

Transcriptome analysis of PBMCs exposed to PM_10_ revealed 14,508 mRNA transcripts, and after filtered mRNAs of absolute value of log_2_ fold change ≥ 1 (FDR < 0.05), 1196 genes (10.6084/m9.figshare.22194115) were observed to be expressed differentially (DEGs), of which 667 (55.8%) and 529 (44.2%) were up and down-regulated, respectively, compared to control cells. To discriminate PBMCs exposed to PM_10_ and control samples, a Principal Component Analysis (PCA) was used (Fig. 1a). High heterogeneity of mRNAs between PBMCs exposed to PM_10_ was observed in the PCA, indicated by the spatial dispersion between samples, suggesting intragroup variability. Different gene expression profiles were observed between control PBMCs and PM_10_-exposed PBMCs using a heat map (Fig. 1b). Moreover, high variability was found between the up-regulated genes in samples of PM_10_-exposed PBMCs (Fig. 1c), mainly in genes involved in immune responses such as *IDO1, IL17F, IFNG, CCL7, CXCL1, CCL17, CD1B, CD1A*, among others. On the other hand, a scatter plot was used to visualize gene expression with the highest degree of regulation (fold change; FC) in the PM_10_-exposed PBMCs compared with the number of transcripts in control (TPM); showing that the highest up-regulated genes were related with the inflammatory response, such as cytokines (I*L1B, IL17F, IL36B, IL36G*, and *TNF*) and chemokines (*CXCL1, CXCL5, CXCL8, CCL7*, and *CCL24*), and the down-regulated genes were related with defense against pathogens, such as antimicrobial peptides (*DEFA3, DEFA4, CAMP* and *LYZ*) and antiviral factors (*APOBEC3A, RNASE1*, and *RNASE6*) (Fig. 1d).

### Genes and biological processes associated with PM_10_ exposure

**Figure 2:**
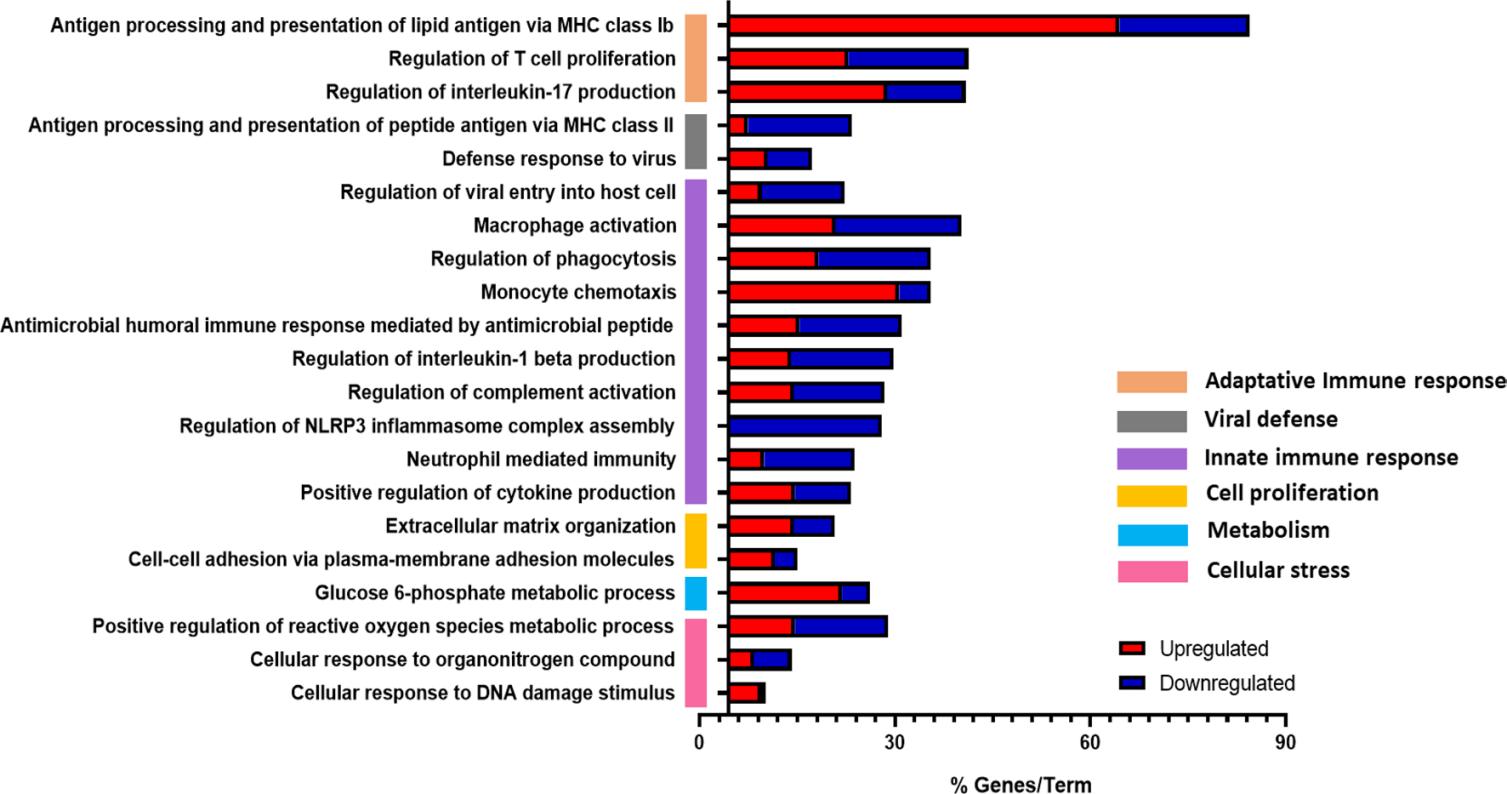
Genes and biological processes associated with PM_10_ exposure. Gene-term enrichment analysis of differentially expressed genes (DEG) in PBMCs exposed to PM_10_. The horizontal bars represent the percentage of genes regulated within the Term (% Genes/Term). Red and blue colors represent up- and down-regulated genes, respectively. The colored-vertical bars represent major gene term groups.

Enrichment analysis was carried out to know the biological processes involved in response to PM_10_ exposure. This gene ontology analysis revealed 27 terms clustered into 6 main groups: adaptive immune response, viral defense, innate immune response, cell proliferation, metabolism, and cellular stress (Fig. 2). Markedly, it was observed the adaptive immune response cluster regulating antigen processing and presentation of lipid antigen via MHC class Ib and MHC class II (Fig. 2). Furthermore, it is observed that the main group of genes regulated are related to innate immune responses, particularly inflammatory responses, with the regulation of monocyte chemotaxis, phagocytosis, complement activation, macrophage activation, and regulation of the NLRP3 complex assembly. In addition, it is notably the up-regulation of the Glucose 6-phosphate pathway (Fig. 2).

Additionally, we explored the highest regulated genes for each term (Figs. S1 and S2). Related to the innate immune response, a higher expression of genes associated with an inflammatory response was observed, such as *SERPINE1, IL1B, CCL7,* and *CCL1.* In addition, lower expression of genes associated with antimicrobial response, such as *CAMP*, *DEFA4*, and *RNASE3* was detected. Genes related to the complement system, such as *C1QC*, *C1QA*, and *C1QB*, also show lower expression levels (Fig. S1). Regarding adaptive immune response genes such as *LGMN*, *HLA-DRB1,* and *PLA2G2D* were down-regulated, while *IL-12B, CD276, SPHK1, CD1B,* and *CD1A* were up-regulated (Fig. S1). For viral defense, lower expression of *APOBEC3A*, *TLR7*, *RNASE6*, *RNASE1*, and *FCN1* was observed in response to PM_10_.

Notably, genes related to other biological processes were up-regulated, such as genes involved in extracellular matrix (*MMP1, MMP10*). Also, it was observed that metabolism-related genes, such as *GPD6, HK2 and HK3;* and genes related to cellular stress, including FPR2, AQP9, CEP63 and CDKN1A, were up-regulated (Fig. S2).

### PM_10_ induces a pro-inflammatory transcriptional profile in PBMCs

**Figure 3:**
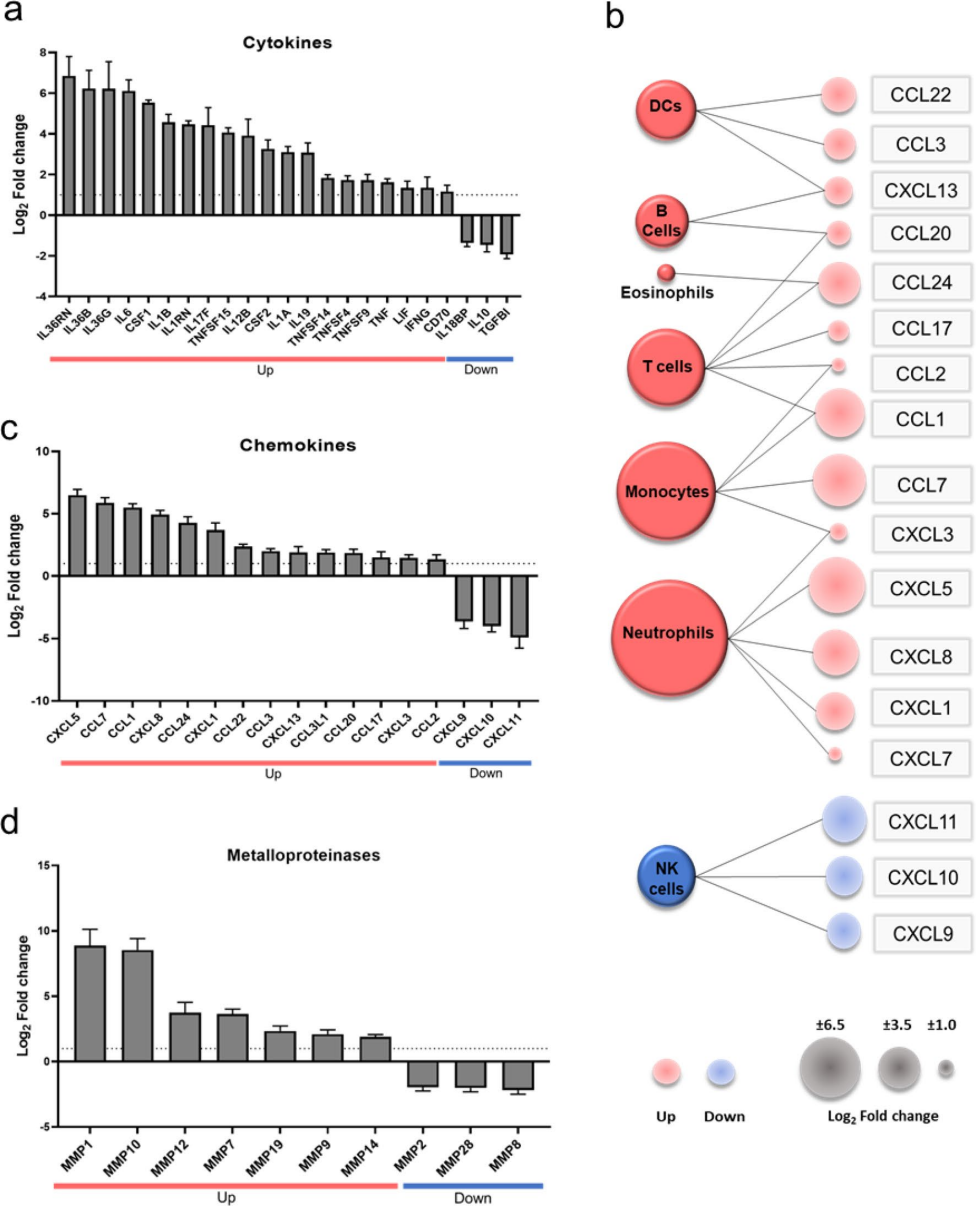
PM_10_ induces a pro-inflammatory transcriptional profile in PBMCs. (**a**) Bar plot with the chemokines regulated by exposure to PM10. (**b**) The cells recruited in response to PM_10_ exposure. Red and blue colors indicate up- and down- regulated genes and dot size indicates the magnitude of log_2_ -fold change. Bar plot of (**c**) cytokines, and (**d**) metalloproteinases regulated during PM10 exposure. Red and blue bars indicate up- and down-regulated genes.

A predominant pro-inflammatory profile was modulated by PM_10_. In this sense, chemokines such as *CXCL5, CCL7, CCL1, and CXCL8* were found to have a FC higher than 4.9. On the contrary, *CXCL9, CXCL10* and *CXCL11* were found to be down-regulated (Fig. 3a). The up-regulated chemokines are mainly related to the recruitment of neutrophils and monocytes, and to a lesser extent, they attract T cells, dendritic cells, B cells, and eosinophils. In the case of negatively regulated chemokines, they are involved in recruiting NK cells (Fig. 3b).

Besides, it was found that the cytokines with a higher FC are from the IL-36, IL-6, and IL-1 family, among others. Additionally, negative regulation of cytokines with anti-inflammatory functions, such as *IL18BP, IL10* and *TGFBI* was observed (Fig. 3c). Likewise, a significant change was found in the expression of metalloproteinases such as MMP1 and *MMP10,* among others (Fig. 3d).

### PM_10_ up-regulates mRNA expression of the IL-36 family members

**Figure 4:**
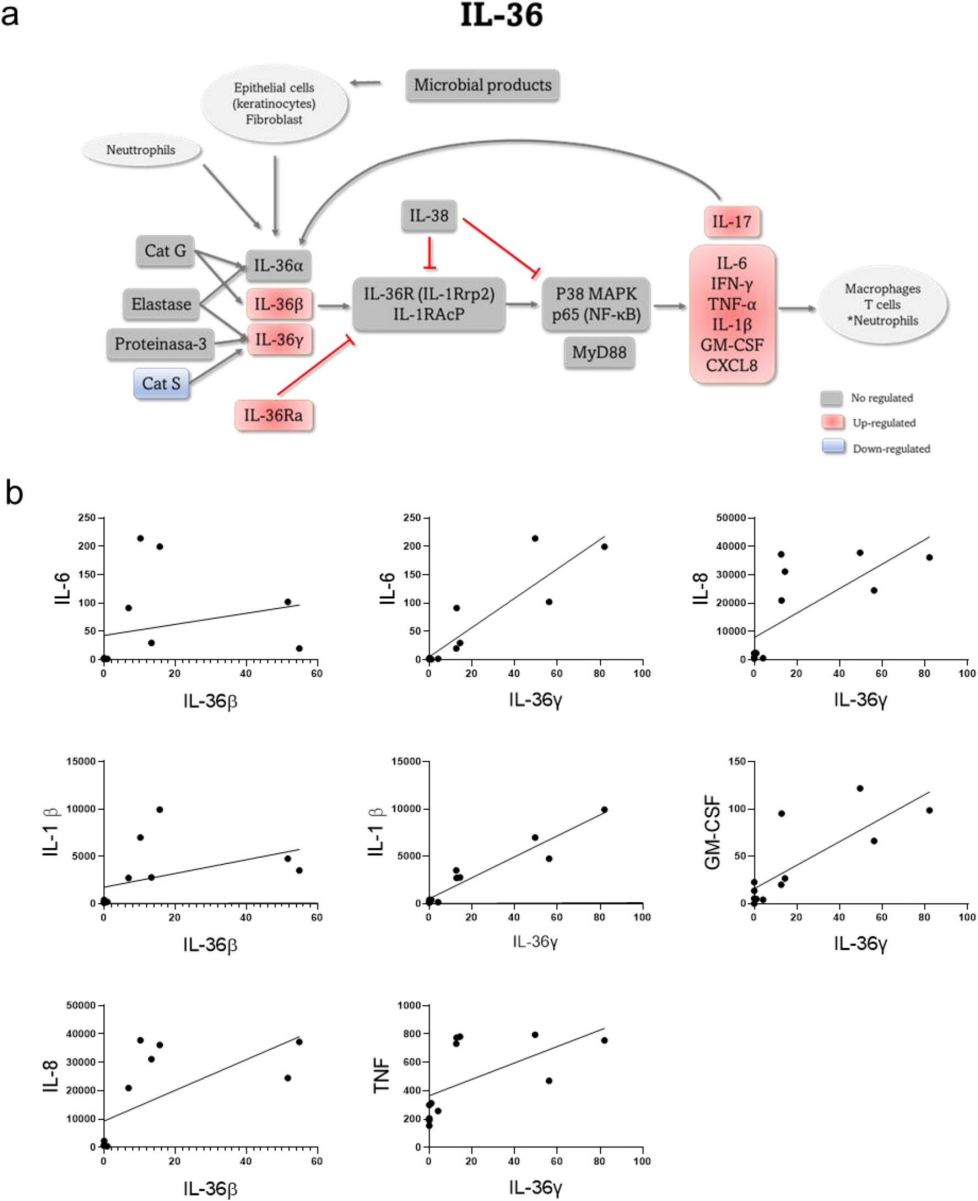
PM_10_ up-regulates mRNA expression of the IL-36 family members. (**a**) Schematic representation of the IL-36 pathway. Red and blue color boxes represent up-and down-regulated genes by PM_10_ exposure. (**b**) Pearson-correlations (r) over 0.74 and p-value < 0.05 were considered a gene co-expression of IL-36 members and IL-1β, IL-6, IL-8, GM-CSF or TNF.

Given that the cytokines with the highest transcriptional regulation induced by PM_10_ were from IL-36 family, the molecules associated with the activation pathway were explored. A positive regulation of IL-36 isoforms (*IL36B* and *IL36G*) and the receptor antagonist (*IL36RN*) was found. Besides, cytokines induced by this pathway were positively regulated, including *IL17*, *IL6*, *IFNG*, *TNF*, *IL1B*, *GMCSF* and *CXCL8* (Fig. 4a). Furthermore, when performing Pearson correlations, a positive correlation was found between *IL36B* and *IL1B* (r = 0.8339, p-value = 0.0015), *IL6* (r = 0.7469, p-value = 0.0079) and *IL-8* (r = 0.7614, p-value = 0.0063); as well as positive correlations between *IL36G* and *IL1B* (r = 0.8791, p-value = 0.0004), *IL-6* (r = 0.8756, p-value = 0.0004), *IL-8* (r = 0.7795, p-value = 0.0043), *GMCSF* (r = 0.7652, p-value = 0.0055) and *TNF* (r = 0.8151, p-value = 0.0021) (Fig. 4b).

### PM_10_ alters the expression of molecules associated with antimicrobial defense

**Figure 5:**
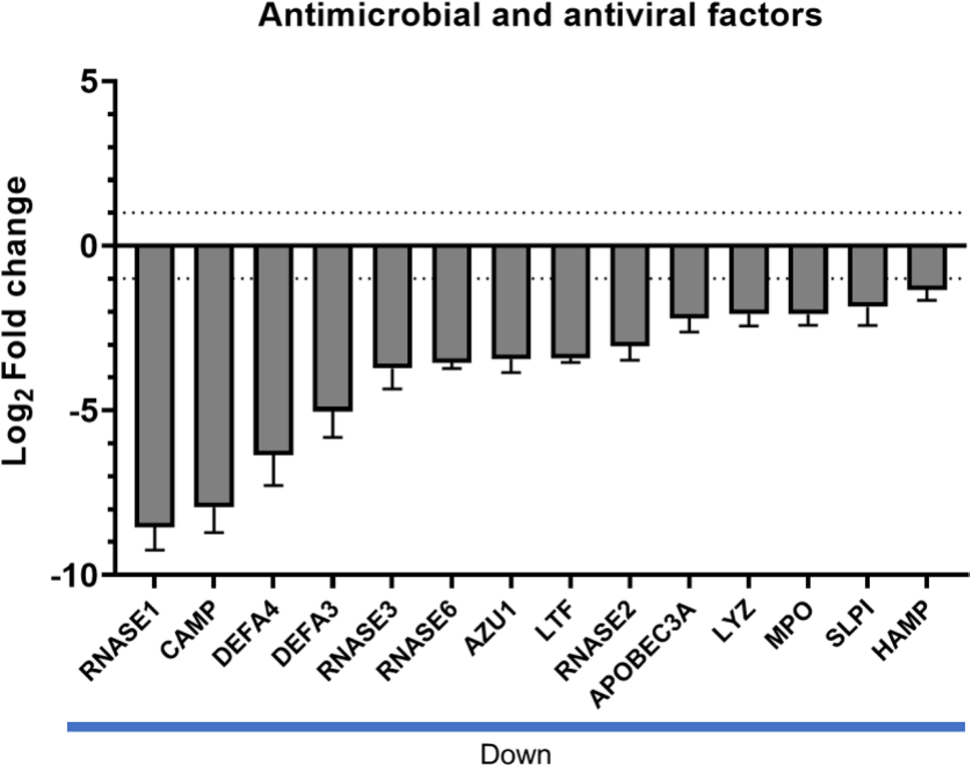
PM_10_ alters the expression of antimicrobial and antiviral factors. The bar plot of antimicrobial and antiviral factors regulated by PM_10_ exposure is represented as log_2_-fold change (y-axis).

Since a negative regulation of molecules associated with defense against pathogens was observed (Fig. 1d), we investigated whether PM_10_ regulated other genes associated with these functions. Negative regulation of antimicrobial and antiviral factors such as *RNASEs, CAMP, DEFAs, AZU1, APOBEC3A, LYZ, and MPO,* among others, was evidenced (Fig. 5).

In addition, upregulation of MHC genes, such as *CD1A, CD1B* and *CD1E,* was observed, as well as negative regulation of *HLA-DQB1, HLA-DMB,* and *HLA-DQA1* (Fig. S3a). On the other hand, negative regulation of components of the complement system was observed, specifically of components 1 (C1) and 2 (C2) genes (Fig. S3b).

### PM_10_ regulates the expression of transcriptional factors and RNA-binding proteins

**Figure 6:**
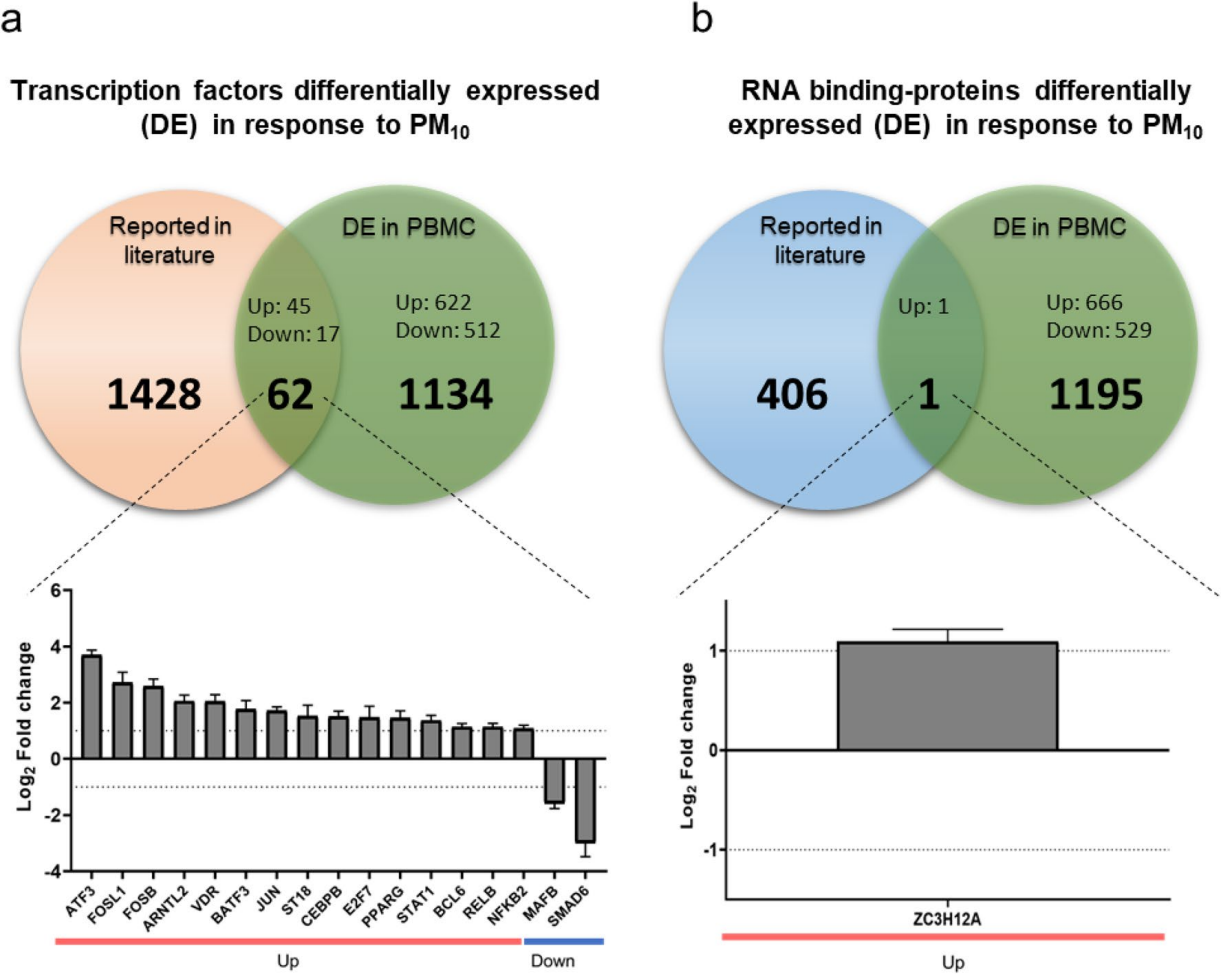
PM_10_ regulates the expression of transcriptional factors and RNA-binding proteins. Bar plots of transcriptional factors (**a**) and RNA-binding proteins (**b**) regulated by PM_10_, represented as log_2_-fold change (y-axis). Red and blue colored-bars indicate up- and down-regulated genes.

Finally, we investigated whether PM_10_ regulates the expression of transcriptional factors. Interestingly, it was found that among DEGs, 62 genes are reported as transcriptional factors and among those with positive regulation are factors associated with inflammatory pathways such as *FOSL1, FOSB, JUN, E2F7, PPARG, RELB* and *NFKB2* (Fig. 6a). On the other hand, *ZC3H12A* was found up-regulated, an RNA-binding protein with endoribonuclease activity, involved in mRNA decay and posttranscriptional gene regulation (Fig. 6b).

## Discussion

Epidemiological and experimental studies have demonstrated the impact of PM exposure on respiratory, cardiovascular, and neurological disorders. Most recently, an increased susceptibility to respiratory viral infections, including SARS-CoV-2, in people exposed to air pollutants was reported^2,4,5,29^. Various detrimental effects on health are attributed to PM, which are related to the induction of prolonged inflammatory and oxidative responses^30^. Previously, the physicochemical characterization of the PM10 sample was carried out, finding that it is mainly composed of ash. In addition, the presence of chemical elements such as Si > Fe > K > Na > Al > Cr > Pt was detected^31^. Particularly, chromium (Cr) and the presence of Benz[a]anthracene, a polycyclic aromatic hydrocarbon (PAH) have been related to the induction of several reactive metabolic intermediates that cause oxidative stress, a process that has been directly related to the inflammatory response^32,33^. This work aimed to characterize the transcriptional response of PBMCs exposed to PM_10_ using RNA-seq to identify mechanisms contributing to the reported deleterious effects**.**

Differential expression of 1196 genes (DEGs) after exposure to PM_10_ was found, mainly related to the inflammatory response, with upregulation of cytokines and chemokines (IL36, IL1B, IL-6, CXCL1, CXCL8, among others). Additionally, downregulation of genes involved in the defense against pathogens was observed, such as antimicrobial peptides (AMPs) and antiviral factors (for example, CAMP, DEFA3, LIZ, MPO). Although few transcriptional analyses in PBMCs exposed to PM_10_ have been reported, a study in Poland reported similar results to ours, describing the regulation of 513 genes in PBMCs of healthy donors after 3 h of exposure to PM_10_, most of them associated with the inflammatory response^34^. In vitro studies also support these findings; inflammatory mediators were produced by a respiratory epithelial cell line exposed to PM_10_^35^. On the other hand, it is interesting to highlight that our analyses revealed downregulation of different genes related to defense against pathogens, especially antimicrobial peptides, suggesting that PM_10_ may affect defense mechanisms. These results suggest that exposure to PM10 for a longer time can induce an inflammatory response, compromising the antimicrobial immune response. These mechanisms could explain the relationship between PM_10_ exposure and greater susceptibility to respiratory infections such as those caused by Influenza, RSV, and SARS-CoV-2, among others^29^. Furthermore, it was observed that PM_10_ induce a response in peripheral blood cells, when recruited to the respiratory epithelium in response to the stimulus, which may contribute to adverse effects^11^.

As expected, the main biological processes involved in DEGs include innate and inflammatory responses. In this sense, it has been described that sub-chronic exposure to low doses of PM_10_ induces an inflammatory response with the recruitment of macrophages and lymphocytes to the lung, increased production of cytokines such as IL-1β and an oxidative state, which have adverse effects in the lungs function^30^. According to this, a positive regulation of transcription factors involved in inflammatory response was found in this study, including ATF3, NFKB2, JUN, FOS, and RELB, among others. ATF3 is induced by stress and plays a role in immunity. Moreover, it has been observed an increase in their expression in response to the activation of Toll-like receptors^36^. However, a dual role of ATF3 has been reported, as evidence shows that it can activate or inhibit proinflammatory cytokines^37–39^. Our study observed increased ATF3 and inflammation, suggesting that ATF3 could promote inflammatory genes. Besides, a positive regulation of the Regulatory binding protein (RBP) ZC3H12A was found. This endoribonuclease negatively regulates inflammation by modulating the IL-6 mRNA stability^40^. Thus, despite the imbalance in the PM-induced immune response, regulatory mechanisms are activated to counteract its pro-inflammatory effects. Nevertheless, it is necessary to carry out studies with prolonged PM exposure times, to elucidate the role of regulators such as ZC3H12A during chronic PM exposure.

It has been described that oxidative stress is one of the main mechanisms triggered by PM exposure. In line with this, we previously observed that PM10 can induce ROS production after 2 h of exposure^31^. In this regard, it has been suggested that this early production of ROS may be an important factor in the induction of cytotoxicity and inflammation in response to PM^41^. Although we found that the main processes regulated in PBMCs at 48 h of exposure to PM10 are related to the inflammatory response, the positive regulation of some oxidative stress genes such as TXN, TXNRD1, NCF2 and NCF4 was observed (Table S1). These results suggest that although the oxidative state is an early event that can induce damage in epithelial and immune cells, it can also contribute to the later effects of PM in conjunction with a strong inflammatory response. However, it would be interesting to perform time kinetics to determine the variation in the expression of oxidative and inflammatory genes, and their association in response to PM10.

Despite that the general response to PM among individuals is pro-inflammatory, the PCA revealed heterogeneity in the transcriptional response triggered by PM_10_ on PBMCs from different healthy donors, compared to control cells, which grouped more homogeneously. Furthermore, when inquiring about the DEGs with the highest coefficient of variation, it was found that these are mainly related to the immune response, including IDO1, CXCL1, CCL17, IL17F, IFNG, and MMP12. Specifically, it can be observed that the PBMCs from donors 5 and 6, exhibited a higher positive regulation of the MT1E and TNIP3 genes and a low gene expression of the chemokines CCL17, CXCL1 and CCL7 (involved in the recruitment of T cells, neutrophils and monocytes, respectively) compared to the donors 2, 3 and 4. TNIP3 is involved in the negative regulation of NF-κB, which seems consistent with the low expression of the inflammatory genes observed. These results suggest differences in the magnitude of the inflammatory response triggered by PM_10_ among individuals, leading to the question of whether these immunogenetic differences could influence the development and exacerbation of diseases related to air pollutants exposure, at individual level^42^.

Additionally, positive regulation of the glucose 6-phosphate pathway was found, indicating that PM_10_ can alter metabolism in PBMCs. In particular, the upregulation of hexokinases 2 and 3 (HK2 and HK3), enzymes that give rise to glucose 6-phosphate, a precursor in glycolysis and biosynthetic pathways such as the pentose phosphate pathway. In addition to their role in generating cellular energy, it has been described that HKs can regulate immune processes such as inflammation^43^. In this regard, Wolf et al. found that the dissociation of HK2 from the mitochondrial membrane induced by peptidoglycan leads to an accumulation of mitochondrial DNA and the production of IL-1β and IL-18 dependent on the NLRP3 inflammasome in macrophages^43^. In this context, it can be argued that exposure to PM_10_ leads to increased glycolysis mediated mainly by HK2 and HK3 in PBMCs, supplying the energy demand that cells require to carry out their functions. However, on the other hand, when faced with infection by Gram-positive bacteria, such as *Bacillus anthracis* and *Staphylococcus aureus,* the up-regulation of HK2 can lead to IL-1β and IL-18 increased production, mediated by the NLRP3 inflammasome most likely inducing an exacerbated inflammatory response.

When delving into the cytokines that exhibited greater up-regulation, the IL-36 subfamily was revealed. Specifically, we found a significant increase in the expression of IL36B, IL36G and IL36RN, cytokines that previously had not been reported to be regulated upon PM_10_ exposure. An important role of these IL-1 family members, in the inflammatory response has been described, being associated with different diseases such as psoriasis and dermatitis, as well as with bacterial and viral infection^44^. It has been described that after binding to its receptor, the recruitment of MYD88 via IRAK, and NF-κB activation is induced, leading to transcription of genes involved in DC maturation, chemotaxis, macrophage and T-cell polarization, and cytokine production, among others^44^. Furthermore, when the regulation of the factors associated with IL-36 signaling and processing pathways in response to PM_10_ was explored, we found regulation of the transcription factor NF-κB, as well as downstream cytokines, such as IL-1β, IL-6, IL-8, and GM-CSF. Moreover, a significant positive correlation was observed between the transcripts of two IL36 isoforms and target pro-inflammatory cytokines, such as IL1B, IL6, IL8, and CSF2, among others, indicating co-expression. Regarding the IL-36 function, both protective and harmful effects of IL-36 have been described. For instance, it has been reported that IL-36 participates in the immune defense against *Citrobacter rodentium*^45^. However, in psoriasis, IL-36 induces the activation of the IL13/IL17A axis at the skin, contributing to the inflammatory process and, thus, to the severity of the disease^46^. Moreover, it has been described that exposure to PM increases the risk of psoriasis flares^47^, suggesting that exposure to PM induces the expression of IL-36, that in turn contributes to the chronic inflammatory response in those patients. Additionally, positive feedback with other pro-inflammatory cytokines has been reported. For example, IL-36α has the capacity to induce the activation signals for the NLRP3 inflammasome in renal tubular epithelial cells, macrophages and DCs, thus favoring the production of IL-1β and IL-18, which in turn may induce more IL-36. This positive feedback leads to an exacerbated inflammatory response that damages the renal tubules and can even lead to kidney failure^48^. Although no other studies have associated the production of IL-36 and the activation of the NLRP3 inflammasome, it would be important to investigate whether this relationship could contribute to inflammatory processes in other pathologies related to PM exposure.

On the other hand, a finding to highlight is the negative regulation of genes encoding for AMPs and antiviral factors. AMPs are components of the innate immune response produced by epithelial cells and phagocytic cells, with antimicrobial functions against microorganisms such as bacteria, fungi, and viruses^49^. It has been described that most AMPs exert their antimicrobial function through membrane permeabilization. However, some can interfere with internal cellular processes, such as the synthesis of macromolecules^50^. Specifically, this study found a negative regulation of CAMP, DEFAs, AZU1, LYZ, and MPO, among others. Although few studies have investigated the effect of PM on the expression of AMPs, Zhang et al., conducted a study in preschool-age children in which they found a negative correlation between chronic exposure to PM_2.5_ and levels of the AMP airway salivary agglutinin (SAG). In addition, they reported a positive correlation between PM_2.5_ levels and peripheral blood cell count, as well as with IL-8 and TNF-α concentrations^51^. In line with these results, a study carried out in BALB/c mice, found that chronic exposure to PM decreases the expression of human β-defensins in the lungs, indicating an alteration in the defense mechanisms in the airways^17^.

Our findings, consistent with previous reports, indicate that exposure to PM decreases the production of antimicrobial peptides in epithelial cells and PBMCs recruited to exposure sites. The alteration of the defense mechanisms against pathogens occurs along with an exacerbated inflammatory response, which trigger the susceptibility to pathogens and the exacerbation of infectious respiratory diseases. In line with this, an in vitro study described that the A549 respiratory epithelial cell line exposed to PM produces lower levels of B-defensins 2 and 3 in response to *Mycobacterium tuberculosis*, as well as increased production of IL-8 and MCP-1, and a higher bacterial load^52^. Even more, although the mechanisms associated with the decrease in the expression of AMPs mediated by PM have not been described, a study carried out in epithelial cells, found that PM exposure increases the invasion by *Pseduomonas aeruginosa* associated with altered secretion of human β-defensin-2 (hBD-2). In addition, they found that PM increases the production of reactive oxygen species (ROS) in response to bacterial infection, accelerated cellular senescence, and decreased hBD-2 production, effects that were attenuated when carrying out treatment with N-acetylcystein, a ROS inhibitor. Furthermore, a positive regulation of IL-8 expression and a negative regulation of IL-13 was observed^14^. These results suggest that exposure to PM alters the expression of AMPs, mediated by ROS induction and the alteration of cytokines such as IL-8, weakening the defense against pathogens.

Moreover, Tatsuta et al. reported that Calu cells exposed to cigarette smoke showed increased cell permeability, with a decrease in genes encoding for adhesion molecules such as claudins, occludin, E-cadherin, and ZO-1, among others. These effects were counteracted in cells pre-treated with LL37 before the exposure to cigarette smoke. Specifically, they observed that pretreatment with these AMPs attenuated the decrease in occludin, offsetting the disruption of these unions^53^. This study highlights the importance of AMPs in the correct airway epithelial barrier function and, hence greater resistance to pathogens, which can be altered by exposure to air pollutants. On the other hand, it has been described that PM can adsorb cationic AMPs, decreasing their availability in the epithelium, and thus contributing to an increase in the microbial load^54^.

Even more, in this study, the downregulation of different genes associated with the complement system was found, something that has not been previously reported. These molecules are involved in the defense against pathogens through opsonization, lysis, and anaphylatoxin release^55^. Likewise, it was observed that HLA-II genes were negatively regulated, which could impair antigen presentation. In this regard, an association has been described between HLA-II and increased risk of developing severe forms of rheumatoid arthritis, in smokers. However, no studies have investigated the direct effect of PM exposure on the expression of HLA-II genes^56^. These results show PM_10_ alters antiviral and antimicrobial factors and could also affect the complement system and antigen presentation, which impairs the development of immune responses. Together, our results suggest that PM exposure worsens airway antimicrobial activity, increasing susceptibility to infections, through mechanisms that need to be deeply explored.

However, additional studies are needed to delve into the genes induced in each cell population and how their expression dynamics change over time. This could be done using a single-cell RNAseq analysis after PM exposure. Further research must be focused on studying proteins and functional activity to identify genes with significant potential to be intervened to reduce the adverse effects observed in response to air pollution.

## Methods

### Preparation of peripheral blood mononuclear cells

Peripheral blood mononuclear cells (PBMCs) were isolated from 6 healthy volunteers (n = 6, males between the ages of 20 and 42). A total of 30 mL of peripheral blood was obtained by venipuncture, with EDTA. Volunteers undergoing long-term medications, smokers, or users of illicit drugs were excluded from study participation. PBMCs were isolated by density gradient method using Ficoll-Histopaque (Sigma-Aldrich Chemical Co., St. Louis, MO, USA) and centrifuged at 2300 rpm for 23 min. Cells were resuspended in complete culture RPMI 1640 medium (Sigma-Aldrich Chemical Co., St. Louis, MO, USA), counted in a hemocytometer, and adjusted to the required concentrations for the various experiments. The viability of PBMCs was 98–100% by trypan blue exclusion in all experiments.

### PM_10_ collection and in vitro exposure of PBMCs

Urban PM_10_ was collected in *Valle de Aburrá*, Colombia, through the local early warning system (Sistema de Alerta Temprana SIATA) of the Valle de Aburrá. Briefly, PM_10_ was collected with high-volume samplers from 100 quartz filters at ten BAM-1020 monitoring stations (TISCH Environmental, BM2000H). Filters were cut into small pieces, high-purity sterile water was added, and stock suspensions were prepared by sonication for 2 h and 15 min at 37 Hz. The mixture was then filtered and lyophilized^31^. PM_10_ was further diluted in sterile water to final concentrations of 100 µg/mL and used to expose in vitro PBMCs for 48 h.

### RNA-Seq analysis

Total RNA was extracted using the Direct-zol RNA MiniPrep kit (Zymo Research, Orange, CA, USA) according to the manufacturer’s instructions. RNA concentration/purity were determined by spectrophotometry and agarose gel electrophoresis. For the mRNA sequencing library, six untreated and six PM_10_ treated samples were constructed with TruSeq Stranded mRNA Sample Prep Kits (Illumina, USA). Samples were indexed with adaptors and submitted for pair-end sequencing using a HiSeq 4000 instrument (Illumina, USA). mRNA libraries were sequenced with a sequencing depth of at least 20 million reads per sample. RNAseq data were analyzed as described before^57^. Then genes were considered differentially expressed if there was an absolute value of log_2_ fold change ≥ 1; and the FDR ≤ 0.05. For assessing mRNA transcript abundance, reads were converted to transcripts per million (TPM).

### Gene enrichment analysis

Enrichr tool was used for gene enrichment analysis with the Gene Ontology database, Kyoto Encyclopedia of Genes and Genomes (KEGG)^58^, and Reactome. The enrichment result is represented as a combined score computed by Enrichr. Also, The Human Protein Atlas database was used to access the functional information for secretome and transcription factors analysis. Finally, we used the database of RNA-binding protein specificities (RBPDB) to access the information on RNA-binding proteins^59^.

### Ethics

All experiments were carried out following the principles of the Declaration of Helsinki. Donors were adults, read and signed and informed consent, previously reviewed, and approved by the research ethics committee of the Universidad Cooperativa de Colombia (Act 001-2022).

### Informed consent

Informed consent was obtained from all the participants and/or their legal guardians.

### Consent to participate

Informed consent was obtained from all subjects involved in the study.

### Supplementary Information

Supplementary Figures.

Supplementary Table 1.

## Data Availability

All data generated or analyzed during this study are included in this published article [and its supplementary information files]. Dataset is avalaible at: https://doi.org/10.6084/m9.figshare.22194115.v1.The datasets generated for this study can be found in the Gene Expression Omnibus (GEO) DataSets (https://www.ncbi.nlm.nih.gov/gds) under the accession numbers GSE226707.
