# Supplementary material for: Particulate matter impairs immune system function by up-regulating inflammatory pathways and decreasing pathogen response gene expression
Source: Sci Rep. 2023 Aug 7;13:12773. doi: 10.1038/s41598-023-39921-w (PMC10406897; doi:10.1038/s41598-023-39921-w)
Supplement: Supplementary file 1 — Supplementary Figures. [file 41598_2023_39921_MOESM1_ESM.docx]

**Supplementary figures**


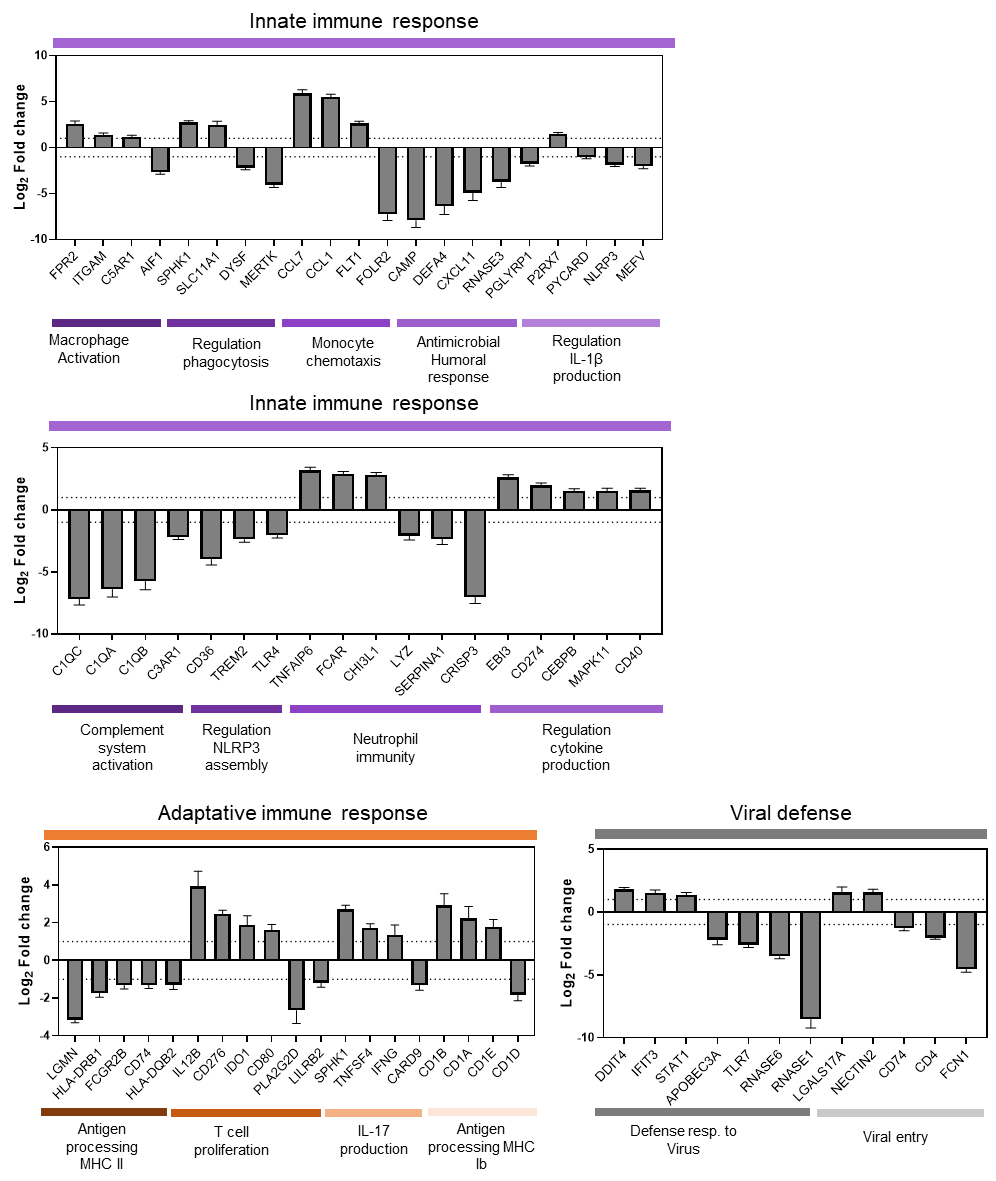


**Figure S1. Primary genes regulated by PM_10_ exposure within each term related to immune response.** Barplot of DEG selected for each term, to show regulated genes in PBMCs exposed to PM represented as log_2_-fold change (y-axis).


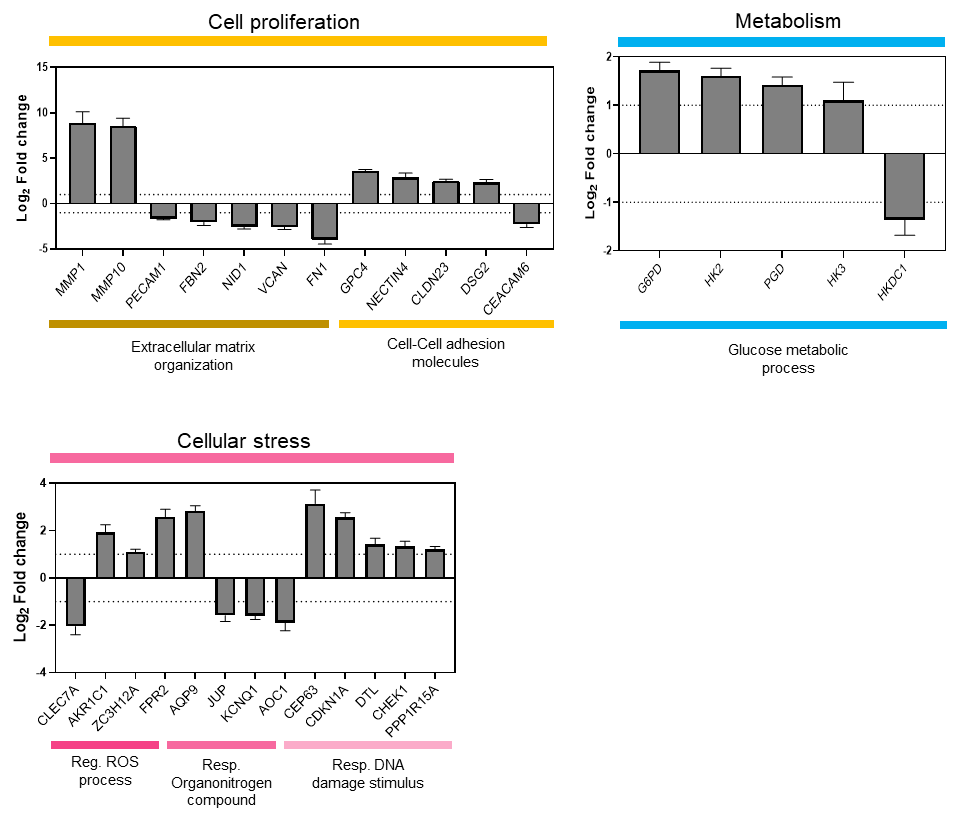


**Figure S2. Primary genes regulated by PM_10_ exposure, from different biological processes.** Barplot of DEG selected for each term, to show the most regulated genes in PBMCs exposed to PM represented as log_2_-fold change (y-axis).


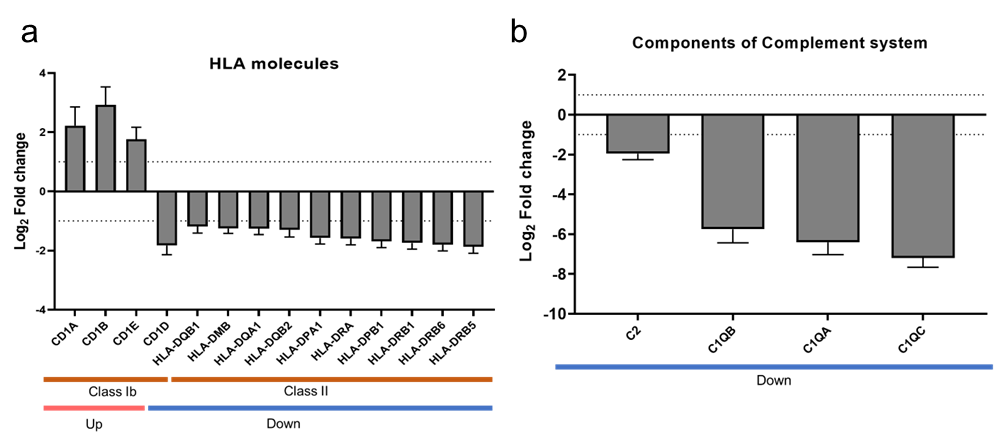


**Figure S3. PM_10_ alters the expression of HLA-related molecules and the complement system.** Bar plot of HLA molecules **(a)**, complement system and **(b)** and Molecules related to pathogens, represented as log_2_-fold change (y-axis). Red and blue colored-bars indicate up- and down-regulated genes.
