# Supplementary material for: Particulate matter impairs immune system function by up-regulating inflammatory pathways and decreasing pathogen response gene expression
Source: Sci Rep. 2023 Aug 7;13:12773. doi: 10.1038/s41598-023-39921-w (PMC10406897; doi:10.1038/s41598-023-39921-w)
Supplement: Supplementary file 2 — Supplementary Table 1. [file 41598_2023_39921_MOESM2_ESM.docx]

**Differentially expressed genes in response to PM10**

| **Gene** | **Log2 Fold Change** | **p-Value adjusted** |
| --- | --- | --- |
| MMP1 | 8.8821 | 1.55E-11 |
| MMP10 | 8.5414 | 1.09E-20 |
| CA12 | 7.2488 | 2.61E-59 |
| INHBA | 7.1932 | 2.27E-87 |
| IL36RN | 6.8537 | 1.05E-11 |
| DPYSL3 | 6.7387 | 6.03E-121 |
| MET | 6.7300 | 4.04E-84 |
| CXCL5 | 6.4936 | 2.19E-42 |
| IL36B | 6.2305 | 6.17E-11 |
| IL36G | 6.2203 | 3.04E-05 |
| IL6 | 6.1135 | 1.72E-27 |
| TM4SF1 | 6.0938 | 1.91E-11 |
| PDLIM4 | 6.0604 | 3.58E-12 |
| GREM1 | 6.0533 | 2.05E-147 |
| ATP1B2 | 6.0306 | 2.39E-76 |
| SERPINB7 | 6.0010 | 4.11E-10 |
| CCL7 | 5.8750 | 2.79E-43 |
| LRTM2 | 5.7867 | 4.83E-13 |
| ITGB3 | 5.7361 | 6.19E-39 |
| CSF1 | 5.5368 | 0 |
| CCL1 | 5.5051 | 5.78E-71 |
| OCSTAMP | 5.4234 | 1.64E-17 |
| ANKRD1 | 5.3751 | 1.55E-80 |
| SPINK1 | 5.1943 | 1.21E-70 |
| SERPINE1 | 5.1680 | 5.93E-85 |
| RPLP0P2 | 4.9761 | 8.31E-32 |
| CXCL8 | 4.9343 | 3.40E-43 |
| LINC00520 | 4.9141 | 1.99E-13 |
| LINC02154 | 4.8423 | 2.08E-16 |
| INHBA-AS1 | 4.7083 | 7.31E-07 |
| AK4 | 4.5819 | 7.54E-68 |
| IL1B | 4.5745 | 2.18E-30 |
| EREG | 4.5427 | 4.41E-34 |
| IL1RN | 4.4687 | 1.01E-138 |
| MT1G | 4.4259 | 7.33E-06 |
| IL17F | 4.4244 | 3.94E-06 |
| C22orf42 | 4.3254 | 1.07E-05 |
| HCAR2 | 4.2987 | 2.23E-43 |
| HCAR3 | 4.2771 | 1.44E-26 |
| CCL24 | 4.2768 | 2.79E-17 |
| OVOL1 | 4.2326 | 3.36E-07 |
| OLIG2 | 4.1905 | 3.72E-09 |
| NCS1 | 4.1444 | 5.14E-76 |
| TREML3P | 4.1264 | 2.44E-10 |
| TMEM158 | 4.1105 | 1.81E-22 |
| EMILIN1 | 4.1100 | 3.90E-72 |
| MYO1B | 4.1062 | 2.29E-56 |
| ITGB8 | 4.0812 | 1.12E-44 |
| TNFSF15 | 4.0671 | 5.35E-66 |
| IL12B | 3.9110 | 1.57E-05 |
| SLC1A2 | 3.9026 | 1.17E-31 |
| PHF24 | 3.7839 | 4.62E-09 |
| MMP12 | 3.7484 | 1.92E-05 |
| KRT79 | 3.7023 | 2.98E-09 |
| ATF3 | 3.6970 | 1.44E-97 |
| CXCL1 | 3.6925 | 3.55E-09 |
| GEM | 3.6877 | 1.65E-21 |
| LOC100128059 | 3.6851 | 4.35E-09 |
| GAL | 3.6765 | 4.62E-05 |
| ALDH1A2 | 3.6747 | 1.37E-33 |
| MMP7 | 3.6529 | 3.64E-21 |
| FAM124A | 3.6516 | 1.03E-09 |
| S1PR3 | 3.6235 | 7.02E-08 |
| BMP6 | 3.6196 | 1.76E-21 |
| KREMEN1 | 3.6076 | 4.36E-31 |
| CTTN | 3.6071 | 6.05E-69 |
| CYP1A1 | 3.5973 | 1.84E-10 |
| GPC4 | 3.5931 | 2.41E-114 |
| SLC28A3 | 3.5566 | 3.92E-31 |
| LOC107984450 | 3.5542 | 1.04E-08 |
| SERPINB2 | 3.5420 | 2.41E-20 |
| WNT5A | 3.5231 | 1.76E-13 |
| G0S2 | 3.5224 | 1.21E-36 |
| ASPHD1 | 3.5183 | 1.15E-18 |
| ROR1-AS1 | 3.4819 | 1.74E-10 |
| SLC7A11-AS1 | 3.4725 | 4.33E-10 |
| MSC | 3.3945 | 7.75E-60 |
| LOC100507403 | 3.3904 | 2.59E-21 |
| TGM2 | 3.3869 | 5.68E-58 |
| LPAR4 | 3.3694 | 3.19E-11 |
| LINC01426 | 3.3671 | 2.00E-09 |
| TSKU | 3.3488 | 3.56E-26 |
| RGS20 | 3.3325 | 3.14E-06 |
| ZNF697 | 3.3219 | 1.71E-46 |
| TCHH | 3.2683 | 7.94E-08 |
| CSF2 | 3.2579 | 5.07E-12 |
| C15orf48 | 3.2508 | 2.75E-48 |
| RAI14 | 3.2480 | 6.95E-30 |
| MAS1 | 3.2350 | 5.82E-07 |
| TNIP3 | 3.1921 | 1.98E-07 |
| FERMT2 | 3.1917 | 3.89E-12 |
| TNFAIP6 | 3.1811 | 7.30E-33 |
| SLC7A11 | 3.1660 | 1.33E-89 |
| PTGS2 | 3.1618 | 2.99E-29 |
| NANOS1 | 3.1579 | 7.93E-07 |
| TRPA1 | 3.1470 | 1.19E-07 |
| CEP63 | 3.1359 | 8.91E-07 |
| EGR2 | 3.1347 | 5.87E-85 |
| MSC-AS1 | 3.1344 | 2.73E-52 |
| IL1A | 3.0966 | 4.52E-27 |
| RASAL2-AS1 | 3.0786 | 4.27E-05 |
| F3 | 3.0759 | 2.10E-20 |
| IL19 | 3.0754 | 2.04E-09 |
| VSTM4 | 3.0649 | 3.95E-06 |
| MYBPH | 3.0363 | 2.16E-06 |
| CCNA1 | 3.0199 | 5.79E-14 |
| DCSTAMP | 3.0064 | 8.01E-30 |
| ELOVL7 | 3.0026 | 3.94E-11 |
| PTGR1 | 2.9963 | 6.11E-55 |
| CYP27B1 | 2.9704 | 1.63E-31 |
| SLC6A9 | 2.9677 | 2.17E-12 |
| MYL9 | 2.9513 | 1.66E-12 |
| CD1B | 2.9197 | 2.18E-05 |
| MME | 2.9182 | 1.82E-21 |
| PAPLN | 2.9074 | 6.93E-33 |
| FCAR | 2.9003 | 4.12E-52 |
| ZMIZ1-AS1 | 2.8979 | 1.78E-104 |
| TMEM217 | 2.8806 | 2.51E-09 |
| NECTIN4 | 2.8675 | 1.03E-07 |
| PDGFA | 2.8620 | 1.10E-24 |
| EPOP | 2.8578 | 4.95E-32 |
| SDC2 | 2.8404 | 2.10E-46 |
| AQP9 | 2.8285 | 2.95E-36 |
| SLC41A2 | 2.8267 | 1.31E-42 |
| C1QTNF1 | 2.8150 | 1.93E-05 |
| THBD | 2.8000 | 4.87E-29 |
| CHI3L1 | 2.7992 | 7.88E-37 |
| RAP1GAP | 2.7911 | 8.89E-10 |
| KCP | 2.7618 | 3.81E-36 |
| ZBTB7C | 2.7469 | 5.26E-06 |
| CD109 | 2.7457 | 2.85E-29 |
| BCAR1 | 2.7375 | 1.01E-06 |
| FOSL1 | 2.7212 | 1.56E-12 |
| PWRN1 | 2.7137 | 0.008488999 |
| KIAA0319 | 2.7137 | 1.33E-10 |
| LINC01010 | 2.7114 | 3.53E-26 |
| SPHK1 | 2.7091 | 2.52E-34 |
| ESPL1 | 2.7071 | 3.27E-43 |
| PLVAP | 2.6880 | 6.04E-09 |
| TNFRSF12A | 2.6723 | 9.66E-15 |
| AICDA | 2.6689 | 1.52E-08 |
| ARMC9 | 2.6624 | 2.70E-40 |
| TREM1 | 2.6529 | 8.31E-11 |
| AK8 | 2.6512 | 2.71E-17 |
| MGST1 | 2.6488 | 2.06E-37 |
| MAFF | 2.6435 | 2.21E-67 |
| A4GALT | 2.6372 | 1.28E-19 |
| LINC01551 | 2.6346 | 0.026833073 |
| NRIP3 | 2.6298 | 2.98E-35 |
| MT1H | 2.6244 | 0.000576147 |
| FLT1 | 2.6240 | 6.53E-26 |
| HSD11B1 | 2.6239 | 6.28E-06 |
| EBI3 | 2.6217 | 4.05E-31 |
| MEP1A | 2.6180 | 7.66E-07 |
| TACSTD2 | 2.6122 | 8.72E-12 |
| PLAUR | 2.6107 | 5.83E-82 |
| MYOF | 2.5995 | 5.44E-41 |
| MCEMP1 | 2.5899 | 2.65E-17 |
| LONRF2 | 2.5812 | 8.10E-05 |
| FOSB | 2.5803 | 5.19E-21 |
| SHF | 2.5763 | 1.07E-26 |
| FPR2 | 2.5754 | 4.68E-14 |
| ANKRD63 | 2.5718 | 0.003161924 |
| SLCO4A1 | 2.5672 | 1.06E-38 |
| CDKN1A | 2.5562 | 3.27E-34 |
| VWCE | 2.5148 | 8.22E-09 |
| AMZ1 | 2.4911 | 5.35E-32 |
| RASAL2 | 2.4910 | 3.22E-42 |
| SPOCD1 | 2.4838 | 2.66E-13 |
| CLDN23 | 2.4829 | 2.63E-32 |
| NPR1 | 2.4820 | 4.81E-09 |
| SLC11A1 | 2.4772 | 7.54E-09 |
| ASAP2 | 2.4743 | 2.85E-09 |
| CD276 | 2.4684 | 8.00E-39 |
| EMP1 | 2.4678 | 8.39E-62 |
| LINC01192 | 2.4658 | 0.012043345 |
| LOC100419170 | 2.4619 | 2.83E-12 |
| PHLDA2 | 2.4605 | 2.55E-05 |
| LINC03025 | 2.4578 | 8.77E-05 |
| SLC12A8 | 2.4486 | 1.07E-16 |
| CHAC1 | 2.4403 | 2.64E-09 |
| KCNE1 | 2.4238 | 4.50E-09 |
| CNKSR3 | 2.4224 | 0.000161927 |
| SOCS3 | 2.4085 | 2.62E-31 |
| FLRT2 | 2.4060 | 8.54E-19 |
| TXN | 2.3740 | 6.54E-50 |
| TXNRD1 | 2.3736 | 1.35E-78 |
| TIE1 | 2.3724 | 3.22E-14 |
| CCL22 | 2.3680 | 1.21E-33 |
| TFRC | 2.3417 | 3.00E-48 |
| CECR2 | 2.3405 | 9.13E-07 |
| MMP19 | 2.3392 | 1.73E-08 |
| TJP1 | 2.3352 | 0.000320164 |
| DSG2 | 2.3141 | 3.81E-11 |
| PHLDA1 | 2.3130 | 5.92E-52 |
| HBEGF | 2.3088 | 2.47E-25 |
| TMEM38B | 2.2982 | 6.12E-26 |
| LINC01694 | 2.2908 | 7.47E-05 |
| DNAJB5 | 2.2791 | 1.31E-24 |
| MOB3B | 2.2773 | 8.36E-08 |
| ATP13A3 | 2.2761 | 8.87E-29 |
| MT1E | 2.2748 | 8.46E-06 |
| NOTCH3 | 2.2337 | 1.39E-26 |
| CD1A | 2.2229 | 0.002791078 |
| LINC02345 | 2.2182 | 3.44E-05 |
| NAMPTP1 | 2.2094 | 2.47E-14 |
| IGDCC4 | 2.1855 | 0.006700692 |
| DUSP4 | 2.1814 | 3.59E-29 |
| PROS1 | 2.1735 | 0.001041588 |
| DYRK3 | 2.1703 | 7.51E-15 |
| CLEC5A | 2.1696 | 7.08E-29 |
| BAALC | 2.1675 | 3.14E-13 |
| IER3 | 2.1546 | 5.26E-26 |
| KYNU | 2.1499 | 2.34E-39 |
| MFSD12 | 2.1455 | 2.40E-54 |
| SH3BP5 | 2.1376 | 9.48E-35 |
| CLDN12 | 2.1337 | 3.45E-19 |
| NAMPT | 2.1324 | 3.66E-45 |
| LRRTM4 | 2.1288 | 0.026553842 |
| MT2A | 2.1202 | 3.93E-06 |
| NMRAL2P | 2.1166 | 4.40E-19 |
| RSAD2 | 2.1127 | 2.14E-28 |
| MAMLD1 | 2.1092 | 3.05E-07 |
| ASPH | 2.1074 | 6.00E-53 |
| GRIK2 | 2.1006 | 0.034158841 |
| TFPI | 2.0984 | 1.02E-08 |
| MMP9 | 2.0969 | 2.59E-09 |
| MSANTD3 | 2.0966 | 3.95E-34 |
| CLEC6A | 2.0882 | 0.000364874 |
| ADGRG2 | 2.0874 | 5.35E-05 |
| CYP1B1 | 2.0686 | 9.35E-28 |
| ARNTL2 | 2.0584 | 2.01E-20 |
| SCARF1 | 2.0511 | 5.54E-41 |
| KCNN4 | 2.0496 | 2.05E-21 |
| VDR | 2.0487 | 1.58E-16 |
| CCND1 | 2.0448 | 2.15E-17 |
| CYGB | 2.0285 | 6.37E-09 |
| KCNJ1 | 2.0149 | 6.43E-09 |
| STAC | 2.0104 | 0.018017395 |
| HSPB7 | 2.0037 | 0.021490535 |
| DUSP5 | 1.9992 | 5.12E-28 |
| SSTR2 | 1.9957 | 0.000119009 |
| SORT1 | 1.9932 | 1.08E-17 |
| CCL3 | 1.9917 | 2.72E-20 |
| NALF1 | 1.9851 | 0.022686258 |
| CD274 | 1.9833 | 1.44E-22 |
| ZNF366 | 1.9716 | 6.71E-19 |
| FCRL4 | 1.9690 | 0.000232788 |
| TM4SF19-AS1 | 1.9534 | 3.05E-09 |
| NR4A3 | 1.9534 | 1.35E-24 |
| LAMB3 | 1.9518 | 8.72E-12 |
| CRYM | 1.9443 | 0.001224706 |
| RGS1 | 1.9416 | 2.62E-29 |
| SHOX2 | 1.9372 | 7.98E-08 |
| KIF14 | 1.9369 | 6.78E-05 |
| CREB5 | 1.9328 | 2.60E-16 |
| TRIM71 | 1.9258 | 0.018316073 |
| STRIP2 | 1.9257 | 3.09E-09 |
| GADD45G | 1.9247 | 3.37E-09 |
| ARRDC4 | 1.9230 | 2.93E-22 |
| AKR1C1 | 1.9163 | 7.81E-08 |
| EVC | 1.9159 | 2.37E-10 |
| ENTHD1 | 1.9147 | 8.02E-05 |
| PSAT1 | 1.9136 | 1.35E-34 |
| SPSB1 | 1.9129 | 2.81E-27 |
| TM4SF19 | 1.9072 | 1.34E-08 |
| ADGRE3 | 1.9047 | 4.28E-07 |
| BCL2L14 | 1.9036 | 0.003072747 |
| MGAT5B | 1.9025 | 6.17E-11 |
| MMP14 | 1.8994 | 2.23E-31 |
| FCER2 | 1.8989 | 4.06E-34 |
| SLC16A6 | 1.8966 | 1.90E-21 |
| RIMS2 | 1.8941 | 0.04930254 |
| CXCL13 | 1.8904 | 0.000525556 |
| ACSL1 | 1.8861 | 5.53E-26 |
| CCL3L1 | 1.8849 | 1.94E-13 |
| HSD3B7 | 1.8821 | 1.23E-13 |
| IDO1 | 1.8766 | 0.00083991 |
| PANX2 | 1.8742 | 1.28E-07 |
| GJB2 | 1.8716 | 0.000962756 |
| GJA3 | 1.8667 | 0.004102702 |
| DLGAP5 | 1.8634 | 2.44E-07 |
| CCL20 | 1.8603 | 4.10E-09 |
| NIBAN2 | 1.8593 | 2.51E-21 |
| ALCAM | 1.8533 | 5.15E-30 |
| KCNN3 | 1.8532 | 0.001057018 |
| GLDC | 1.8418 | 6.59E-05 |
| NEDD4L | 1.8404 | 3.27E-26 |
| TNFSF14 | 1.8304 | 1.94E-27 |
| HPD | 1.8240 | 0.000979318 |
| CTNNAL1 | 1.8235 | 5.37E-20 |
| DDIT4 | 1.8081 | 1.34E-37 |
| CKB | 1.8065 | 2.56E-14 |
| BCAT1 | 1.8034 | 1.83E-13 |
| TMEM132A | 1.8021 | 5.82E-14 |
| MIR100HG | 1.8006 | 0.009991939 |
| SLC7A5 | 1.7992 | 5.32E-53 |
| BEGAIN | 1.7973 | 0.014740918 |
| ARRDC3-AS1 | 1.7955 | 1.99E-05 |
| SNX10-AS1 | 1.7808 | 0.00072921 |
| PRKAG3 | 1.7772 | 0.000525432 |
| KEL | 1.7753 | 1.41E-05 |
| P4HA2 | 1.7751 | 1.24E-05 |
| BATF3 | 1.7739 | 8.26E-08 |
| CD1E | 1.7653 | 0.000117871 |
| SOX5 | 1.7644 | 0.004333481 |
| GPAT3 | 1.7591 | 4.55E-24 |
| COL8A2 | 1.7574 | 1.86E-10 |
| CYP7B1 | 1.7407 | 0.00027467 |
| EPIC1 | 1.7342 | 0.041680713 |
| ALAS1 | 1.7322 | 7.88E-13 |
| TNFSF4 | 1.7290 | 4.62E-15 |
| G6PD | 1.7234 | 3.69E-24 |
| PPP2R3A | 1.7225 | 3.06E-10 |
| JUN | 1.7221 | 2.35E-37 |
| CYP1B1-AS1 | 1.7219 | 1.37E-10 |
| TNFSF9 | 1.7217 | 2.38E-08 |
| CCRL2 | 1.7198 | 2.23E-24 |
| SLC12A5-AS1 | 1.7191 | 0.000127429 |
| LOC100288175 | 1.7174 | 4.53E-17 |
| CSRP2 | 1.7092 | 0.019251104 |
| RGS16 | 1.7045 | 3.78E-08 |
| PYCR1 | 1.6970 | 3.25E-09 |
| FNIP2 | 1.6964 | 2.99E-15 |
| FAIM | 1.6946 | 5.33E-13 |
| SPRY2 | 1.6899 | 1.42E-13 |
| PRRG1 | 1.6858 | 0.002687105 |
| RAB38 | 1.6809 | 8.37E-07 |
| LOC284191 | 1.6796 | 0.000111092 |
| PTPRF | 1.6776 | 4.06E-05 |
| ICAM5 | 1.6766 | 9.02E-06 |
| ITGA2B | 1.6750 | 6.07E-06 |
| CDCP1 | 1.6743 | 3.03E-11 |
| CAVIN1 | 1.6725 | 0.000144233 |
| NPTX1 | 1.6725 | 5.66E-05 |
| ATP6V1FNB | 1.6725 | 7.05E-06 |
| LUCAT1 | 1.6706 | 5.86E-37 |
| USP2 | 1.6681 | 0.010038639 |
| TSPAN33 | 1.6668 | 1.19E-11 |
| GDF15 | 1.6632 | 1.22E-09 |
| CRIM1 | 1.6532 | 2.80E-17 |
| DIAPH3 | 1.6507 | 0.001401936 |
| CES1 | 1.6505 | 0.004499407 |
| SNX10 | 1.6372 | 2.56E-11 |
| DAGLA | 1.6372 | 1.96E-25 |
| WARS1 | 1.6344 | 4.39E-14 |
| CDH1 | 1.6335 | 0.000183972 |
| SRC | 1.6204 | 6.04E-23 |
| PACSIN3 | 1.6200 | 0.002642872 |
| TNF | 1.6172 | 4.30E-20 |
| ITGA1 | 1.6152 | 1.73E-10 |
| SLC24A3 | 1.6151 | 0.000351283 |
| GK | 1.6133 | 1.44E-13 |
| SPINT1 | 1.6066 | 1.63E-23 |
| CD80 | 1.6062 | 6.41E-07 |
| HK2 | 1.6026 | 2.29E-21 |
| BCL2A1 | 1.6011 | 3.22E-14 |
| MCM10 | 1.6009 | 0.000101914 |
| KIF26B | 1.5957 | 1.28E-05 |
| KCNJ11 | 1.5955 | 3.33E-06 |
| LAMP3 | 1.5933 | 1.23E-37 |
| SORBS1 | 1.5932 | 4.76E-06 |
| NAT8L | 1.5902 | 0.026973572 |
| CDC45 | 1.5883 | 5.23E-05 |
| CRABP2 | 1.5850 | 5.58E-06 |
| UBTD1 | 1.5835 | 4.91E-11 |
| MAP1B | 1.5827 | 0.038521415 |
| ZBED2 | 1.5825 | 9.50E-09 |
| PRKAG2-AS2 | 1.5802 | 2.13E-09 |
| LRG1 | 1.5747 | 1.81E-07 |
| KIF18B | 1.5726 | 0.000187577 |
| CD40 | 1.5722 | 3.47E-16 |
| STAP2 | 1.5712 | 0.0012836 |
| PSD3 | 1.5705 | 1.92E-08 |
| SDC4 | 1.5681 | 2.11E-13 |
| NECTIN2 | 1.5648 | 7.16E-10 |
| ADGRE2 | 1.5618 | 6.86E-19 |
| LGALS17A | 1.5586 | 0.001770059 |
| DNAJC6 | 1.5571 | 1.03E-08 |
| NCF2 | 1.5469 | 9.90E-17 |
| ZFYVE16 | 1.5443 | 1.08E-13 |
| H1-2 | 1.5407 | 8.02E-11 |
| LPL | 1.5392 | 1.16E-13 |
| GBP6 | 1.5341 | 0.016949146 |
| STX11 | 1.5314 | 6.11E-21 |
| ST18 | 1.5311 | 0.000427784 |
| CDC25A | 1.5281 | 7.13E-05 |
| CIR1 | 1.5249 | 0.00010253 |
| CDKN3 | 1.5222 | 4.52E-05 |
| LRP12 | 1.5205 | 4.60E-13 |
| IFIT3 | 1.5173 | 3.42E-09 |
| MTHFD2 | 1.5144 | 2.27E-20 |
| TNFRSF9 | 1.5140 | 1.94E-13 |
| FSCN1 | 1.5139 | 3.22E-15 |
| CD83 | 1.5113 | 1.81E-31 |
| MCOLN3 | 1.5071 | 4.35E-05 |
| CEBPB | 1.5068 | 5.26E-13 |
| MAPK11 | 1.5038 | 3.05E-08 |
| TTC28 | 1.4967 | 1.02E-11 |
| CCL17 | 1.4965 | 0.005929334 |
| TNFRSF18 | 1.4951 | 1.24E-12 |
| NEK6 | 1.4945 | 3.34E-20 |
| IQGAP3 | 1.4932 | 2.15E-05 |
| RAPGEF5 | 1.4883 | 0.000945093 |
| TRIP13 | 1.4870 | 1.43E-06 |
| EXO1 | 1.4812 | 1.52E-05 |
| APBB2 | 1.4801 | 4.26E-07 |
| ETS2 | 1.4781 | 2.33E-35 |
| PHLDB1 | 1.4752 | 1.03E-06 |
| TMEM119 | 1.4714 | 0.000308233 |
| CD82 | 1.4704 | 3.05E-30 |
| E2F7 | 1.4694 | 0.00196141 |
| RASSF8 | 1.4693 | 6.74E-05 |
| GPER1 | 1.4661 | 0.003697109 |
| CEP112 | 1.4642 | 0.000829887 |
| ASAP1 | 1.4609 | 5.70E-24 |
| PPARG | 1.4604 | 1.28E-07 |
| RGCC | 1.4603 | 1.08E-13 |
| KIF4A | 1.4570 | 0.007462879 |
| ST14 | 1.4551 | 1.03E-12 |
| ADAMTS7 | 1.4548 | 0.009753474 |
| CXCL3 | 1.4492 | 2.11E-07 |
| P2RX7 | 1.4492 | 1.19E-14 |
| RAB13 | 1.4434 | 1.69E-08 |
| CDC6 | 1.4430 | 4.92E-08 |
| KIF1B | 1.4376 | 1.31E-20 |
| HOMER1 | 1.4321 | 5.19E-06 |
| UCHL1 | 1.4306 | 4.07E-07 |
| DHCR24 | 1.4300 | 7.03E-17 |
| ZNF462 | 1.4287 | 0.006657614 |
| PGD | 1.4229 | 2.60E-17 |
| DTL | 1.4205 | 4.72E-07 |
| CFAP58-DT | 1.4185 | 0.000444928 |
| TMEM163 | 1.4177 | 1.12E-08 |
| MACC1 | 1.4173 | 6.74E-05 |
| ADM2 | 1.4159 | 3.24E-10 |
| CDKN2B | 1.4149 | 7.97E-09 |
| SLC1A4 | 1.4142 | 9.18E-30 |
| TDRD6 | 1.4071 | 0.000551184 |
| ITGAM | 1.4061 | 1.59E-13 |
| ADM | 1.4020 | 7.47E-14 |
| PTPRE | 1.4020 | 5.16E-23 |
| MTHFD1L | 1.4003 | 1.61E-15 |
| MIR155HG | 1.3927 | 6.79E-16 |
| B3GNT5 | 1.3884 | 0.01815459 |
| HMMR | 1.3880 | 3.12E-05 |
| SLAMF7 | 1.3871 | 1.89E-15 |
| ANKRD33B | 1.3860 | 3.31E-11 |
| SOCS1 | 1.3792 | 5.80E-06 |
| PMAIP1 | 1.3779 | 1.36E-20 |
| GGH | 1.3757 | 8.89E-07 |
| PDLIM7 | 1.3743 | 3.64E-10 |
| ABL2 | 1.3715 | 5.04E-21 |
| STAT1 | 1.3699 | 8.73E-13 |
| SLITRK4 | 1.3673 | 0.00522782 |
| IRAK2 | 1.3670 | 3.47E-18 |
| MICALL2 | 1.3664 | 5.16E-07 |
| SKA3 | 1.3581 | 0.002272076 |
| LONRF1 | 1.3572 | 5.59E-14 |
| CDC20 | 1.3518 | 0.000590193 |
| CCL2 | 1.3499 | 0.00129168 |
| ZFHX2 | 1.3497 | 0.001798682 |
| IFNG | 1.3469 | 0.043124002 |
| C3 | 1.3458 | 0.000921719 |
| NIBAN1 | 1.3452 | 4.98E-25 |
| NDRG2 | 1.3429 | 1.32E-25 |
| MAP1A | 1.3399 | 7.12E-08 |
| LIF | 1.3388 | 0.000490658 |
| TICRR | 1.3381 | 0.001449526 |
| HMG20B | 1.3373 | 2.48E-14 |
| KLF10 | 1.3320 | 1.74E-16 |
| LINC00996 | 1.3314 | 9.13E-08 |
| TMEM121B | 1.3283 | 1.14E-07 |
| TUBB6 | 1.3274 | 1.13E-11 |
| ABCC1 | 1.3266 | 1.95E-35 |
| HNF1B | 1.3229 | 0.040505414 |
| CHEK1 | 1.3212 | 1.34E-07 |
| GRASLND | 1.3201 | 8.45E-05 |
| SIGLEC15 | 1.3199 | 1.90E-10 |
| DOCK3 | 1.3158 | 6.34E-09 |
| SYP | 1.3154 | 1.03E-05 |
| SNX9 | 1.3130 | 4.91E-11 |
| DUSP1 | 1.3109 | 2.24E-20 |
| MAPK10 | 1.3102 | 0.026648548 |
| CDK14 | 1.3101 | 2.78E-12 |
| CCDC26 | 1.3100 | 0.014839574 |
| CCDC40 | 1.3071 | 0.034581608 |
| H2AC6 | 1.3063 | 1.51E-07 |
| LRRC28 | 1.3026 | 7.14E-07 |
| CRMP1 | 1.2958 | 0.000576972 |
| PCDHGC3 | 1.2958 | 0.003295398 |
| NTRK2 | 1.2948 | 0.031081053 |
| PCSK6 | 1.2933 | 4.85E-05 |
| CYP51A1 | 1.2925 | 0.002138555 |
| DOCK4 | 1.2910 | 3.07E-11 |
| BMP1 | 1.2899 | 4.71E-11 |
| SEMA7A | 1.2889 | 1.77E-17 |
| PHLDA3 | 1.2889 | 0.005614605 |
| E2F8 | 1.2887 | 0.048110014 |
| TOX2 | 1.2860 | 0.000471815 |
| LRRC32 | 1.2836 | 4.67E-06 |
| RFX2 | 1.2817 | 5.93E-11 |
| CAVIN3 | 1.2776 | 0.038217359 |
| QPCT | 1.2772 | 1.83E-07 |
| TFPI2 | 1.2763 | 0.017873968 |
| CKAP2L | 1.2652 | 0.007545344 |
| SGMS2 | 1.2628 | 0.00020013 |
| BCAR3 | 1.2599 | 6.06E-08 |
| SLC3A2 | 1.2598 | 3.32E-21 |
| TMOD1 | 1.2595 | 0.007268666 |
| CD2BP2-DT | 1.2577 | 9.17E-06 |
| NCAM1 | 1.2553 | 3.18E-09 |
| ZNF804A | 1.2506 | 5.07E-06 |
| GSN | 1.2503 | 1.04E-10 |
| FSD1L | 1.2489 | 1.72E-06 |
| BIRC5 | 1.2473 | 0.001751956 |
| PKD1L1 | 1.2467 | 0.003351011 |
| MYO5C | 1.2445 | 0.007739604 |
| CA2 | 1.2404 | 2.08E-09 |
| SQLE | 1.2403 | 3.84E-25 |
| CDK1 | 1.2400 | 0.000362624 |
| SQOR | 1.2327 | 1.34E-09 |
| CLSPN | 1.2320 | 1.13E-07 |
| FLT3 | 1.2308 | 5.64E-06 |
| MYBL2 | 1.2289 | 2.55E-07 |
| EOGT | 1.2259 | 5.37E-06 |
| LAMC1 | 1.2246 | 3.34E-10 |
| MAP4K3-DT | 1.2244 | 1.15E-06 |
| H2BC21 | 1.2239 | 1.93E-06 |
| NDST1 | 1.2155 | 1.26E-12 |
| MREG | 1.2129 | 1.38E-14 |
| HDAC9 | 1.2077 | 5.63E-09 |
| SLC1A3 | 1.2034 | 6.51E-14 |
| PPP1R15A | 1.2029 | 1.84E-21 |
| PLD1 | 1.2029 | 4.90E-08 |
| TRIM16L | 1.2017 | 0.000620722 |
| DOCK6 | 1.1966 | 7.79E-05 |
| RDX | 1.1962 | 9.81E-10 |
| ACOT7 | 1.1958 | 7.61E-10 |
| MKI67 | 1.1932 | 7.78E-07 |
| SSPN | 1.1921 | 0.000447286 |
| MYO10 | 1.1909 | 0.00737309 |
| FGR | 1.1885 | 2.15E-07 |
| TMEM200A | 1.1815 | 0.002251004 |
| SLC6A12 | 1.1786 | 6.50E-05 |
| CYP27A1 | 1.1778 | 5.08E-14 |
| MATK | 1.1750 | 2.07E-10 |
| LINC01547 | 1.1749 | 1.52E-06 |
| C5AR1 | 1.1748 | 6.58E-12 |
| CBLN3 | 1.1733 | 7.94E-06 |
| TNKS1BP1 | 1.1725 | 3.28E-11 |
| SPR | 1.1698 | 0.000547955 |
| ALOX15B | 1.1684 | 9.23E-09 |
| PPFIBP1 | 1.1660 | 5.21E-07 |
| CD70 | 1.1659 | 0.000924798 |
| USP12 | 1.1652 | 3.30E-13 |
| GSR | 1.1646 | 1.55E-14 |
| BRCA2 | 1.1630 | 1.96E-13 |
| STARD8 | 1.1548 | 1.59E-07 |
| KIF18A | 1.1527 | 0.010793701 |
| MIR9-1HG | 1.1516 | 0.002304533 |
| RRM2 | 1.1508 | 0.00011743 |
| PPIF | 1.1485 | 2.48E-21 |
| PKMYT1 | 1.1420 | 0.004275221 |
| GP1BA | 1.1418 | 1.04E-06 |
| PPBP | 1.1416 | 0.002556398 |
| E2F1 | 1.1406 | 1.04E-05 |
| BCL6 | 1.1383 | 1.12E-19 |
| IGHV3-49 | 1.1368 | 0.015730964 |
| RELB | 1.1358 | 6.38E-17 |
| MELK | 1.1357 | 0.003670389 |
| SLC7A1 | 1.1347 | 1.47E-17 |
| TNFRSF4 | 1.1324 | 1.14E-05 |
| LIMK1 | 1.1317 | 5.28E-10 |
| DOT1L | 1.1311 | 5.94E-23 |
| DRAIC | 1.1309 | 0.035001096 |
| PIM3 | 1.1287 | 8.41E-12 |
| UPP1 | 1.1274 | 1.85E-10 |
| C1S | 1.1263 | 1.63E-05 |
| FEZ1 | 1.1248 | 0.000105696 |
| CDCA5 | 1.1239 | 5.35E-05 |
| CENPN | 1.1208 | 3.36E-07 |
| MTRNR2L8 | 1.1154 | 0.005614605 |
| KLF9 | 1.1141 | 2.55E-10 |
| FAM167A | 1.1141 | 0.004174552 |
| ADO | 1.1118 | 4.47E-12 |
| DYNLT2B | 1.1116 | 0.014770182 |
| HLX | 1.1079 | 3.45E-05 |
| NEPRO-AS1 | 1.1079 | 0.000569051 |
| COL4A2 | 1.1070 | 0.000413589 |
| RASGRF1 | 1.1035 | 9.30E-06 |
| HK3 | 1.1032 | 0.015109666 |
| TTLL4 | 1.1017 | 1.34E-12 |
| ABTB2 | 1.1008 | 1.58E-07 |
| MSMO1 | 1.1007 | 1.28E-06 |
| FAM151B | 1.0993 | 0.013387378 |
| DGAT2 | 1.0979 | 0.000276138 |
| SLC43A3 | 1.0968 | 8.49E-15 |
| SOAT1 | 1.0963 | 4.24E-10 |
| SPRED2 | 1.0961 | 4.43E-08 |
| SLC1A5 | 1.0959 | 6.43E-11 |
| H3C6 | 1.0950 | 0.001688077 |
| CSTB | 1.0950 | 4.78E-07 |
| NRP2 | 1.0941 | 1.80E-08 |
| ZC3H12A | 1.0933 | 1.47E-17 |
| JAKMIP2 | 1.0926 | 1.34E-08 |
| LRP8 | 1.0923 | 3.35E-13 |
| TGFA | 1.0915 | 5.76E-06 |
| DLC1 | 1.0890 | 0.013631664 |
| GPR68 | 1.0868 | 2.10E-14 |
| NFKB2 | 1.0852 | 2.80E-21 |
| FSD1 | 1.0825 | 0.002920874 |
| BHLHE40 | 1.0812 | 6.66E-22 |
| MELTF | 1.0805 | 0.000498687 |
| TTK | 1.0786 | 0.021691745 |
| TOP2A | 1.0767 | 0.000496448 |
| CDCA2 | 1.0752 | 0.031397237 |
| RTN2 | 1.0744 | 0.001408131 |
| LOC100996437 | 1.0739 | 1.91E-06 |
| SLC35E4 | 1.0723 | 0.002444014 |
| KIF23 | 1.0713 | 0.002619104 |
| TBC1D8 | 1.0696 | 3.04E-07 |
| RASGEF1B | 1.0685 | 2.64E-08 |
| SGPP2 | 1.0634 | 4.29E-12 |
| ZNF442 | 1.0626 | 0.007631105 |
| PALM2AKAP2 | 1.0601 | 1.43E-16 |
| ITGAX | 1.0588 | 2.91E-11 |
| CCNB2 | 1.0579 | 0.000403937 |
| HIP1 | 1.0575 | 5.17E-13 |
| DGKG | 1.0522 | 0.001359463 |
| ORC1 | 1.0478 | 0.000842584 |
| ZNRF1 | 1.0476 | 1.26E-09 |
| FURIN | 1.0470 | 3.43E-24 |
| SMOX | 1.0446 | 5.97E-05 |
| CDK18 | 1.0445 | 3.65E-05 |
| ZNF267 | 1.0444 | 1.43E-13 |
| L1CAM | 1.0440 | 4.12E-05 |
| PHGDH | 1.0439 | 2.20E-09 |
| OSGIN1 | 1.0431 | 1.52E-06 |
| PLAT | 1.0425 | 0.002155759 |
| POLQ | 1.0425 | 0.000417883 |
| GBP4 | 1.0423 | 0.031519901 |
| KIFC1 | 1.0414 | 0.000958417 |
| GIPC3 | 1.0403 | 6.86E-06 |
| LOC101927745 | 1.0398 | 0.009080586 |
| CASZ1 | 1.0389 | 6.00E-05 |
| TRAF4 | 1.0387 | 4.81E-10 |
| MKLN1-AS | 1.0365 | 0.002880705 |
| ASB2 | 1.0363 | 0.004425302 |
| VPS37C | 1.0362 | 3.46E-08 |
| UBE2C | 1.0288 | 0.014438181 |
| TEKTIP1 | 1.0285 | 0.000579302 |
| NAV2 | 1.0267 | 0.000818913 |
| FGFR1 | 1.0267 | 2.29E-05 |
| KIR2DL4 | 1.0265 | 0.001514418 |
| KBTBD8 | 1.0258 | 1.81E-08 |
| PMEPA1 | 1.0239 | 1.62E-16 |
| ESCO2 | 1.0225 | 0.001956121 |
| VNN3P | 1.0217 | 0.02357992 |
| GTF2IRD1 | 1.0210 | 6.13E-06 |
| AARS1 | 1.0180 | 2.25E-20 |
| CDKN2B-AS1 | 1.0164 | 0.005326358 |
| MYO1C | 1.0151 | 1.30E-11 |
| BTG3 | 1.0151 | 3.08E-09 |
| MAP4K3 | 1.0056 | 1.70E-06 |
| LMNA | 1.0044 | 1.71E-10 |
| ZSWIM4 | 1.0035 | 5.51E-06 |
| PLEKHA8P1 | -1.0008 | 0.000358961 |
| GRN | -1.0026 | 5.32E-05 |
| TRAV8-2 | -1.0036 | 0.001920503 |
| LIPA | -1.0061 | 5.22E-05 |
| RASSF1 | -1.0065 | 1.37E-21 |
| PDGFB | -1.0082 | 3.86E-08 |
| LAMB2 | -1.0109 | 4.65E-05 |
| ARHGEF4 | -1.0111 | 0.014511435 |
| TTC16 | -1.0126 | 1.25E-10 |
| FGFBP2 | -1.0165 | 2.03E-16 |
| RTP5 | -1.0180 | 0.015209919 |
| FBXL16 | -1.0183 | 3.67E-12 |
| NRGN | -1.0198 | 0.005442321 |
| NHLRC4 | -1.0238 | 0.021449156 |
| MSRB2 | -1.0246 | 4.95E-06 |
| RASSF4 | -1.0249 | 1.11E-05 |
| BST1 | -1.0280 | 4.48E-05 |
| PLEKHG5 | -1.0292 | 1.58E-05 |
| TMEM204 | -1.0301 | 2.56E-14 |
| STEAP3 | -1.0303 | 0.000781571 |
| SERPINF2 | -1.0316 | 0.003136458 |
| RNU4-62P | -1.0330 | 0.047990672 |
| ARHGEF10L | -1.0339 | 0.000280161 |
| PYCARD | -1.0340 | 4.85E-07 |
| AP1S2 | -1.0345 | 1.84E-05 |
| ACSL6 | -1.0347 | 2.59E-08 |
| PITPNM2 | -1.0348 | 4.84E-12 |
| CARD16 | -1.0358 | 2.59E-07 |
| DISC1 | -1.0443 | 4.01E-09 |
| GRAMD4 | -1.0447 | 1.69E-14 |
| SEMA4A | -1.0451 | 1.11E-11 |
| CACNA1I | -1.0460 | 1.77E-21 |
| MS4A14 | -1.0468 | 8.03E-05 |
| RASGRP2 | -1.0508 | 2.74E-26 |
| TESC | -1.0509 | 4.48E-05 |
| ERBB3 | -1.0515 | 3.11E-05 |
| SOWAHD | -1.0522 | 0.000111839 |
| SIGLEC7 | -1.0551 | 2.66E-06 |
| COPZ2 | -1.0620 | 0.031818511 |
| DBP | -1.0645 | 4.79E-14 |
| FST | -1.0692 | 0.007652225 |
| CUEDC1 | -1.0721 | 2.55E-06 |
| TCEA3 | -1.0744 | 8.00E-07 |
| FAM13A | -1.0748 | 2.05E-10 |
| CACNA2D2 | -1.0752 | 4.41E-12 |
| CRYZL2P | -1.0757 | 0.020455908 |
| ADRB2 | -1.0759 | 2.39E-13 |
| VPREB3 | -1.0765 | 0.004434426 |
| BLVRB | -1.0790 | 0.00010081 |
| FCRL6 | -1.0808 | 3.16E-15 |
| CTSS | -1.0824 | 4.48E-09 |
| CYP2E1 | -1.0832 | 0.024848118 |
| PHOSPHO1 | -1.0837 | 0.000119043 |
| GRM2 | -1.0846 | 0.030538447 |
| M1AP | -1.0895 | 0.012022212 |
| PDE6G | -1.0917 | 0.005725361 |
| DPEP3 | -1.0928 | 0.005578945 |
| NCALD | -1.0998 | 2.27E-06 |
| RCN3 | -1.1000 | 0.001345593 |
| MEGF6 | -1.1010 | 6.92E-21 |
| HECW2-AS1 | -1.1021 | 0.000263851 |
| HAL | -1.1031 | 0.000591984 |
| PTGIR | -1.1076 | 6.68E-09 |
| KLHL3 | -1.1106 | 1.83E-07 |
| OPHN1 | -1.1121 | 0.001134183 |
| LINC02611 | -1.1125 | 9.64E-09 |
| NAAA | -1.1137 | 1.27E-21 |
| MAF | -1.1165 | 3.78E-15 |
| A2M-AS1 | -1.1194 | 0.006286942 |
| ID3 | -1.1195 | 2.76E-12 |
| TNS1 | -1.1267 | 3.36E-07 |
| TPCN1 | -1.1286 | 2.74E-17 |
| JDP2 | -1.1287 | 7.86E-08 |
| HOMER3 | -1.1315 | 0.00031649 |
| FGD4 | -1.1331 | 1.23E-05 |
| CEBPD | -1.1392 | 7.85E-09 |
| GASK1B-AS1 | -1.1414 | 0.027016708 |
| RAP1GAP2 | -1.1420 | 1.08E-17 |
| SIGIRR | -1.1421 | 1.41E-20 |
| C12orf42 | -1.1424 | 1.24E-05 |
| OSBPL1A | -1.1469 | 2.70E-06 |
| NFXL1 | -1.1470 | 0.000111292 |
| NHSL2 | -1.1477 | 1.71E-17 |
| NTN4 | -1.1490 | 0.042974242 |
| ABI3 | -1.1516 | 3.89E-18 |
| CLCN4 | -1.1538 | 4.67E-05 |
| ETV5 | -1.1538 | 0.000145592 |
| MILR1 | -1.1552 | 3.66E-06 |
| RNF165 | -1.1557 | 0.006769107 |
| ANO5 | -1.1570 | 0.03131691 |
| KRT73 | -1.1588 | 3.78E-05 |
| LTA4H | -1.1623 | 1.82E-12 |
| RAP2B | -1.1644 | 7.40E-13 |
| MARCKS | -1.1706 | 8.79E-09 |
| DNAAF11 | -1.1712 | 0.03262213 |
| WLS | -1.1719 | 0.001150667 |
| RASSF6 | -1.1745 | 0.010713382 |
| SRGAP3 | -1.1750 | 2.52E-05 |
| ANXA9 | -1.1772 | 1.77E-06 |
| DEPDC7 | -1.1801 | 0.001272365 |
| WNT1 | -1.1833 | 0.001612136 |
| HLA-DQB1 | -1.1842 | 2.33E-06 |
| NOD2 | -1.1872 | 7.22E-10 |
| EPS8 | -1.1878 | 9.96E-05 |
| NSG1 | -1.1893 | 6.31E-11 |
| VENTX | -1.1902 | 0.038447527 |
| CX3CR1 | -1.1905 | 4.76E-22 |
| LILRB2 | -1.1917 | 3.77E-06 |
| HAVCR2 | -1.1929 | 2.25E-16 |
| DNAJB13 | -1.1942 | 0.001376009 |
| SPARC | -1.1960 | 3.12E-11 |
| GAS7 | -1.1965 | 2.18E-16 |
| PACSIN1 | -1.1980 | 7.61E-05 |
| KLF7 | -1.1982 | 9.08E-30 |
| SMPDL3A | -1.2053 | 4.88E-05 |
| PCDH1 | -1.2060 | 0.000115943 |
| NLN | -1.2076 | 5.18E-14 |
| MFGE8 | -1.2178 | 1.20E-15 |
| PAX8 | -1.2223 | 0.000261278 |
| EPPK1 | -1.2231 | 0.000236769 |
| TENT5A | -1.2256 | 3.48E-24 |
| SLC1A7 | -1.2311 | 8.64E-07 |
| OTULINL | -1.2334 | 2.98E-17 |
| PI16 | -1.2353 | 4.78E-05 |
| ABCC5 | -1.2378 | 9.84E-17 |
| CCDC170 | -1.2383 | 0.001065023 |
| KDF1 | -1.2414 | 0.006531985 |
| TMC4 | -1.2461 | 0.004315674 |
| FGD2 | -1.2471 | 5.84E-11 |
| MPP1 | -1.2517 | 4.77E-08 |
| CRYL1 | -1.2521 | 4.17E-15 |
| HLA-DMB | -1.2533 | 1.09E-12 |
| PTGFRN | -1.2555 | 0.000134468 |
| DSC2 | -1.2566 | 0.023834628 |
| P2RY1 | -1.2588 | 0.002611005 |
| HLA-DQA1 | -1.2597 | 5.34E-09 |
| KCNMB4 | -1.2597 | 0.000391274 |
| NUPR1 | -1.2602 | 0.004104912 |
| TNFAIP8L2 | -1.2610 | 6.01E-19 |
| PLXDC1 | -1.2624 | 7.26E-11 |
| DPEP2 | -1.2666 | 7.73E-31 |
| LDLRAD3 | -1.2717 | 0.002019923 |
| ABCA13 | -1.2741 | 0.0081473 |
| LINC01857 | -1.2755 | 9.46E-08 |
| LOC101929698 | -1.2784 | 3.44E-06 |
| FCGR1A | -1.2795 | 0.00201949 |
| ITGB2-AS1 | -1.2850 | 7.32E-18 |
| ITGB5 | -1.2856 | 3.88E-05 |
| PALD1 | -1.2866 | 5.18E-05 |
| CNRIP1 | -1.2885 | 0.002283189 |
| PODN | -1.2901 | 0.013290777 |
| CLMN | -1.2922 | 1.14E-13 |
| C14orf132 | -1.2931 | 3.31E-11 |
| SCIMP | -1.2959 | 3.05E-12 |
| RGS18 | -1.2967 | 4.92E-08 |
| TRIM58 | -1.2975 | 0.003436978 |
| NINJ2 | -1.2975 | 5.85E-06 |
| HLA-DQB2 | -1.3003 | 7.57E-07 |
| TSPAN15 | -1.3018 | 6.60E-05 |
| FCGRT | -1.3026 | 1.42E-07 |
| MLC1 | -1.3032 | 1.03E-08 |
| UCP2 | -1.3046 | 2.38E-24 |
| VSIG1 | -1.3050 | 5.18E-15 |
| EPHA1-AS1 | -1.3088 | 0.000166704 |
| CD74 | -1.3097 | 3.28E-11 |
| FCGR2B | -1.3151 | 9.56E-10 |
| CR1 | -1.3153 | 8.44E-16 |
| TRPM6 | -1.3160 | 0.01489633 |
| CD101 | -1.3178 | 7.17E-10 |
| CSF1R | -1.3188 | 4.75E-13 |
| RGS12 | -1.3203 | 1.16E-08 |
| COL5A3 | -1.3213 | 5.95E-09 |
| VSIR | -1.3247 | 4.88E-21 |
| CARD9 | -1.3268 | 3.47E-06 |
| ZMYND10 | -1.3269 | 0.001816718 |
| MDS2 | -1.3348 | 1.60E-05 |
| SLC14A1 | -1.3389 | 1.28E-09 |
| RIN2 | -1.3393 | 3.58E-09 |
| HAMP | -1.3395 | 0.00021211 |
| TLR2 | -1.3426 | 2.48E-19 |
| LCN8 | -1.3450 | 0.006531985 |
| KCNJ2 | -1.3451 | 0.000201391 |
| GOLGA7B | -1.3463 | 1.45E-09 |
| PSAP | -1.3481 | 2.58E-10 |
| LINC02908 | -1.3492 | 2.15E-05 |
| CLEC11A | -1.3535 | 1.11E-05 |
| TRPM2 | -1.3536 | 2.28E-11 |
| AOAH | -1.3536 | 4.84E-18 |
| NUDT16L2P | -1.3597 | 0.008743354 |
| SNX24 | -1.3622 | 3.07E-09 |
| SLC16A5 | -1.3639 | 5.25E-10 |
| IL18BP | -1.3677 | 1.24E-12 |
| HKDC1 | -1.3682 | 0.00012723 |
| LGR4 | -1.3702 | 0.000917087 |
| MYOM2 | -1.3744 | 0.000547173 |
| LINC01730 | -1.3749 | 8.94E-06 |
| TFCP2L1 | -1.3772 | 2.62E-08 |
| PRSS23 | -1.3794 | 1.05E-19 |
| LRRC25 | -1.3843 | 3.04E-14 |
| CPED1 | -1.3850 | 6.31E-05 |
| ZNF704 | -1.3900 | 1.86E-11 |
| PLB1 | -1.3917 | 0.001498334 |
| ACRBP | -1.3920 | 2.01E-09 |
| ARMCX1 | -1.3922 | 7.57E-07 |
| SASH1 | -1.3931 | 4.85E-08 |
| WDR86-AS1 | -1.3949 | 0.005771845 |
| CEACAM3 | -1.4062 | 0.000900766 |
| LINC02273 | -1.4081 | 4.57E-10 |
| BPI | -1.4102 | 4.16E-13 |
| SLC29A1 | -1.4127 | 1.78E-10 |
| PMFBP1 | -1.4172 | 4.20E-06 |
| NPL | -1.4174 | 8.13E-09 |
| CXCR2 | -1.4277 | 2.07E-10 |
| GASK1B | -1.4300 | 0.004527247 |
| SLC37A2 | -1.4347 | 3.38E-12 |
| LXN | -1.4394 | 3.93E-08 |
| HSF4 | -1.4424 | 1.16E-08 |
| NME8 | -1.4453 | 7.88E-10 |
| MARCHF1 | -1.4469 | 2.30E-15 |
| CCDC65 | -1.4477 | 1.44E-14 |
| HS3ST1 | -1.4481 | 4.16E-05 |
| HMOX1 | -1.4527 | 1.33E-09 |
| KRT72 | -1.4581 | 1.74E-16 |
| GPR162 | -1.4590 | 4.63E-06 |
| LDHD | -1.4609 | 0.019802073 |
| FKBP9 | -1.4611 | 2.51E-05 |
| STAC3 | -1.4620 | 2.49E-07 |
| LOC105373780 | -1.4632 | 0.000217828 |
| SLC9A9 | -1.4642 | 1.87E-20 |
| VIPR1 | -1.4650 | 2.23E-20 |
| LRRC4 | -1.4652 | 8.94E-05 |
| IL10 | -1.4653 | 0.000169671 |
| RAB37 | -1.4657 | 6.41E-24 |
| SIRPB1 | -1.4673 | 1.01E-20 |
| LTBP2 | -1.4731 | 1.28E-09 |
| ALOX5 | -1.4802 | 2.18E-14 |
| ADGRG1 | -1.4814 | 5.90E-41 |
| FES | -1.4830 | 6.33E-20 |
| TSPAN32 | -1.4875 | 5.80E-30 |
| SPNS3 | -1.4939 | 0.000239708 |
| OR4D1 | -1.4940 | 2.68E-05 |
| CTSC | -1.4949 | 2.79E-17 |
| DENND2B | -1.4989 | 0.001036123 |
| CNTNAP2 | -1.4989 | 3.18E-09 |
| C1orf162 | -1.5060 | 1.14E-36 |
| C11orf21 | -1.5109 | 2.50E-39 |
| NFAM1 | -1.5206 | 0.000229822 |
| TPM2 | -1.5255 | 1.07E-16 |
| MNDA | -1.5315 | 7.08E-09 |
| PADI2 | -1.5337 | 7.69E-17 |
| KLF2 | -1.5427 | 2.71E-39 |
| DHRS9 | -1.5428 | 4.05E-15 |
| EEPD1 | -1.5441 | 2.00E-07 |
| FCER1A | -1.5467 | 3.27E-06 |
| DAPK1 | -1.5485 | 1.07E-11 |
| REPS2 | -1.5502 | 1.09E-07 |
| NRCAM | -1.5533 | 4.00E-08 |
| ARG1 | -1.5563 | 1.49E-06 |
| CCDC9B | -1.5578 | 8.30E-18 |
| LMO2 | -1.5599 | 3.26E-14 |
| LAIR1 | -1.5599 | 2.68E-29 |
| ADAP2 | -1.5660 | 1.07E-10 |
| CLIC2 | -1.5722 | 7.68E-10 |
| CD302 | -1.5756 | 4.63E-06 |
| HLA-DPA1 | -1.5766 | 2.03E-13 |
| ITSN1 | -1.5845 | 9.96E-11 |
| HRH2 | -1.5848 | 1.47E-16 |
| JUP | -1.5882 | 4.06E-09 |
| TBXAS1 | -1.5953 | 6.99E-33 |
| MAFB | -1.5963 | 1.66E-19 |
| HLA-DRA | -1.5981 | 7.77E-13 |
| AATBC | -1.6013 | 1.09E-07 |
| KCNQ1 | -1.6060 | 2.24E-26 |
| SIGLEC14 | -1.6168 | 2.42E-23 |
| METTL7A | -1.6200 | 6.68E-33 |
| TSPAN18 | -1.6202 | 4.02E-13 |
| LY86 | -1.6391 | 3.74E-17 |
| LGALS1 | -1.6427 | 1.12E-16 |
| ACP3 | -1.6436 | 3.00E-11 |
| IQCD | -1.6519 | 0.001512597 |
| GATM | -1.6529 | 5.60E-16 |
| WNT10B | -1.6544 | 1.08E-14 |
| PECAM1 | -1.6557 | 5.85E-35 |
| ATP8B4 | -1.6576 | 7.15E-12 |
| FOLR3 | -1.6684 | 0.00035683 |
| SFRP5 | -1.6695 | 3.75E-06 |
| C16orf74 | -1.6727 | 5.73E-10 |
| DOK2 | -1.6734 | 1.15E-21 |
| IGFBP7 | -1.6752 | 1.34E-10 |
| MIR223HG | -1.6777 | 3.10E-17 |
| CD177 | -1.6876 | 0.000252386 |
| CTSK | -1.6914 | 3.32E-14 |
| HLA-DPB1 | -1.6933 | 6.54E-15 |
| DEPTOR | -1.6960 | 8.79E-05 |
| ALDH2 | -1.6984 | 2.95E-05 |
| F2RL3 | -1.7033 | 0.002619104 |
| DGKK | -1.7172 | 1.96E-05 |
| PMP22 | -1.7268 | 3.82E-05 |
| AK5 | -1.7271 | 2.60E-17 |
| LACC1 | -1.7325 | 6.66E-27 |
| HSPA6 | -1.7355 | 3.38E-08 |
| LINC01451 | -1.7356 | 0.000203241 |
| GJB6 | -1.7362 | 0.000556618 |
| CPAMD8 | -1.7438 | 5.82E-06 |
| HLA-DRB1 | -1.7445 | 1.34E-15 |
| HTR7 | -1.7490 | 2.25E-06 |
| PGLYRP1 | -1.7512 | 5.85E-10 |
| MS4A3 | -1.7545 | 9.68E-10 |
| NEFL | -1.7558 | 0.000304443 |
| NHS | -1.7594 | 0.00010276 |
| GPA33 | -1.7637 | 7.62E-23 |
| SNCA | -1.7654 | 4.88E-09 |
| GPR141 | -1.7660 | 2.42E-15 |
| PRSS36 | -1.7689 | 3.16E-07 |
| S100A4 | -1.7738 | 2.47E-35 |
| SECTM1 | -1.7744 | 0.001140794 |
| KANK2 | -1.7784 | 5.77E-11 |
| PTGDS | -1.7838 | 0.00048284 |
| GAS6 | -1.7839 | 8.70E-23 |
| MGST2 | -1.7923 | 4.38E-13 |
| OTOA | -1.7940 | 0.002615495 |
| MARVELD1 | -1.7944 | 5.62E-14 |
| HSD17B14 | -1.7946 | 2.36E-08 |
| PID1 | -1.7954 | 4.71E-11 |
| HLA-DRB6 | -1.8027 | 9.30E-17 |
| ANKRD44-AS1 | -1.8212 | 1.51E-06 |
| KRT73-AS1 | -1.8256 | 1.14E-05 |
| CD1D | -1.8275 | 9.17E-08 |
| SLC2A9 | -1.8310 | 1.47E-16 |
| SEMA3A | -1.8324 | 4.11E-06 |
| RAB32 | -1.8349 | 1.98E-13 |
| MAML3 | -1.8437 | 3.35E-16 |
| SLPI | -1.8495 | 0.006524697 |
| NLRP3 | -1.8627 | 5.10E-18 |
| HLA-DRB5 | -1.8670 | 3.32E-15 |
| GPBAR1 | -1.8703 | 6.03E-06 |
| RASGRP4 | -1.8796 | 9.35E-14 |
| ADA2 | -1.8857 | 5.84E-46 |
| AOC1 | -1.8991 | 1.59E-07 |
| ZNF467 | -1.9060 | 1.81E-13 |
| FCGR3A | -1.9070 | 2.54E-34 |
| HNMT | -1.9148 | 1.43E-15 |
| IGF1 | -1.9315 | 0.009040006 |
| TGFBI | -1.9326 | 2.49E-18 |
| NMUR1 | -1.9356 | 2.79E-18 |
| ARPIN | -1.9373 | 3.70E-19 |
| LINC02432 | -1.9491 | 0.000388531 |
| CD180 | -1.9514 | 5.35E-32 |
| CFP | -1.9519 | 6.64E-20 |
| MYCL | -1.9554 | 1.10E-21 |
| FPR3 | -1.9560 | 0.000105049 |
| C2 | -1.9560 | 1.84E-09 |
| TNNT1 | -1.9595 | 0.004421261 |
| NCF4 | -1.9636 | 9.77E-29 |
| FGL2 | -1.9705 | 4.48E-16 |
| MMP2 | -1.9706 | 6.26E-12 |
| PDGFC | -1.9805 | 3.96E-20 |
| DSC1 | -1.9806 | 3.06E-05 |
| CYBRD1 | -1.9953 | 2.15E-12 |
| WNT7A | -1.9960 | 1.05E-16 |
| LRMDA | -2.0025 | 2.97E-05 |
| MMP28 | -2.0123 | 3.72E-10 |
| IL1R2 | -2.0200 | 5.83E-05 |
| MEFV | -2.0222 | 2.41E-11 |
| SLC24A4 | -2.0305 | 3.96E-07 |
| CD4 | -2.0456 | 2.63E-56 |
| SRPX | -2.0459 | 0.000577751 |
| MS4A7 | -2.0466 | 1.01E-05 |
| TLR4 | -2.0498 | 2.72E-20 |
| CLEC7A | -2.0550 | 6.09E-08 |
| CEACAM8 | -2.0564 | 2.77E-18 |
| FBN2 | -2.0604 | 2.23E-07 |
| LYZ | -2.0743 | 9.85E-08 |
| ASRGL1 | -2.0773 | 8.63E-15 |
| MPO | -2.0849 | 3.10E-09 |
| MSR1 | -2.0854 | 0.000747882 |
| DNASE2 | -2.0979 | 2.99E-37 |
| ORM1 | -2.0988 | 5.97E-08 |
| TCN2 | -2.1081 | 5.47E-16 |
| MMP8 | -2.1891 | 1.55E-11 |
| C3AR1 | -2.1922 | 2.92E-25 |
| CRACDL | -2.1992 | 4.57E-09 |
| APOBEC3A | -2.2035 | 1.70E-06 |
| STEAP4 | -2.2111 | 0.001254357 |
| RAB42 | -2.2132 | 3.38E-13 |
| GPR82 | -2.2133 | 1.77E-10 |
| DYSF | -2.2228 | 2.63E-35 |
| IGSF10 | -2.2375 | 0.003348799 |
| CCDC149 | -2.2553 | 3.76E-16 |
| RGL1 | -2.2590 | 1.79E-26 |
| CEACAM6 | -2.2865 | 1.02E-09 |
| SLC8A1 | -2.2932 | 4.66E-19 |
| LINC02009 | -2.2968 | 0.019729619 |
| MPP3 | -2.2983 | 0.000142352 |
| KCNJ10 | -2.3263 | 8.99E-07 |
| LOC105369519 | -2.3362 | 8.24E-09 |
| SERPINA1 | -2.3489 | 9.94E-07 |
| TREM2 | -2.3781 | 1.01E-23 |
| KLHDC8B | -2.3884 | 7.17E-30 |
| KCTD12 | -2.3933 | 1.63E-10 |
| TMEM86A | -2.3989 | 1.17E-18 |
| SCNN1A | -2.4080 | 2.33E-06 |
| SERPINF1 | -2.4092 | 4.60E-24 |
| TCN1 | -2.4444 | 5.21E-14 |
| AXL | -2.4522 | 9.60E-10 |
| CEACAM4 | -2.4550 | 1.52E-06 |
| LINC01259 | -2.4581 | 4.22E-13 |
| COL23A1 | -2.4663 | 1.26E-23 |
| LOC728488 | -2.5199 | 2.85E-06 |
| NID1 | -2.5427 | 1.41E-21 |
| CXCR1 | -2.5674 | 3.78E-15 |
| LINC01503 | -2.5689 | 5.57E-12 |
| TREML1 | -2.5707 | 3.59E-08 |
| VCAN | -2.6009 | 1.52E-20 |
| TLR7 | -2.6038 | 8.50E-28 |
| ENPP2 | -2.6207 | 9.19E-06 |
| PLA2G2D | -2.6512 | 0.000866329 |
| AIF1 | -2.6789 | 1.64E-30 |
| RHOBTB1 | -2.6807 | 1.68E-12 |
| HP | -2.6904 | 1.95E-13 |
| APBA1 | -2.6973 | 5.34E-27 |
| NAIP | -2.7003 | 6.06E-24 |
| TMEM176B | -2.7297 | 1.27E-09 |
| TMEM176A | -2.7352 | 2.87E-11 |
| CMKLR1 | -2.7460 | 6.48E-48 |
| GAPT | -2.8119 | 6.43E-48 |
| SIRPB2 | -2.8258 | 3.14E-35 |
| SLC46A1 | -2.8265 | 1.87E-36 |
| P2RY13 | -2.8317 | 4.84E-18 |
| ISM1 | -2.8423 | 5.92E-05 |
| FABP4 | -2.9197 | 7.94E-08 |
| STARD13 | -2.9356 | 2.05E-19 |
| ALK | -2.9465 | 1.27E-07 |
| PDK4 | -2.9909 | 2.49E-14 |
| SMAD6 | -2.9942 | 1.41E-08 |
| MPEG1 | -3.0108 | 1.60E-41 |
| CFD | -3.0509 | 2.65E-35 |
| RNASE2 | -3.0574 | 4.84E-12 |
| GSDMA | -3.0950 | 2.14E-07 |
| SDC3 | -3.1124 | 1.39E-07 |
| CST3 | -3.1132 | 1.20E-11 |
| CHST13 | -3.1181 | 4.07E-09 |
| TSPAN4 | -3.1225 | 2.38E-41 |
| TLR5 | -3.1296 | 1.12E-38 |
| CCR2 | -3.1336 | 1.57E-39 |
| LGMN | -3.1586 | 1.73E-92 |
| CSF3R | -3.1851 | 8.22E-39 |
| GFRA2 | -3.2241 | 6.67E-17 |
| STK32B | -3.2612 | 1.56E-07 |
| APOC1 | -3.2715 | 1.72E-06 |
| APOE | -3.2759 | 6.22E-05 |
| FCGR3B | -3.3134 | 1.40E-13 |
| P2RY6 | -3.3255 | 9.75E-37 |
| STAB1 | -3.3312 | 2.55E-53 |
| OTOAP1 | -3.3435 | 6.96E-09 |
| FXYD6 | -3.3562 | 1.37E-12 |
| SDS | -3.3635 | 2.65E-26 |
| NRG1 | -3.3746 | 0.001732868 |
| RAB3IL1 | -3.4108 | 7.95E-05 |
| GPR34 | -3.4167 | 1.67E-40 |
| LTF | -3.4202 | 1.02E-158 |
| GPNMB | -3.4368 | 5.67E-16 |
| AZU1 | -3.4462 | 6.90E-16 |
| VMO1 | -3.4701 | 3.74E-11 |
| FZD2 | -3.4839 | 2.47E-35 |
| RNASE6 | -3.5494 | 7.59E-84 |
| CD300E | -3.5735 | 3.37E-23 |
| RBP7 | -3.6140 | 3.18E-09 |
| RBP1 | -3.6205 | 3.31E-09 |
| CXCL9 | -3.6335 | 1.22E-09 |
| TIFAB | -3.6355 | 1.15E-14 |
| PKD2L1 | -3.6416 | 2.45E-10 |
| CTTNBP2 | -3.6550 | 4.05E-15 |
| KCNMA1 | -3.7000 | 1.66E-10 |
| MYO7A | -3.7237 | 1.17E-23 |
| RNASE3 | -3.7293 | 3.32E-08 |
| C1orf127 | -3.7388 | 4.48E-11 |
| ASGR1 | -3.7508 | 3.38E-18 |
| EPHB2 | -3.7993 | 3.07E-55 |
| SLC47A1 | -3.8462 | 2.94E-32 |
| CD14 | -3.8899 | 5.55E-117 |
| ANOS1 | -3.8937 | 9.30E-08 |
| HGF | -3.9166 | 1.88E-31 |
| CLEC10A | -3.9610 | 1.04E-12 |
| GPRC5B | -3.9617 | 2.29E-07 |
| SLCO2B1 | -3.9714 | 2.46E-104 |
| CD36 | -3.9854 | 7.68E-16 |
| CXCL10 | -4.0017 | 3.36E-16 |
| FN1 | -4.0121 | 4.76E-17 |
| KCNJ5 | -4.0237 | 1.17E-42 |
| MERTK | -4.0396 | 1.82E-38 |
| SELENOP | -4.0457 | 5.83E-13 |
| FUCA1 | -4.1356 | 2.00E-73 |
| MARCO | -4.2141 | 8.40E-31 |
| MS4A4A | -4.3002 | 1.59E-15 |
| ALDH1A1 | -4.3216 | 4.15E-96 |
| OLFM1 | -4.3264 | 2.91E-08 |
| TMIGD3 | -4.3485 | 2.39E-13 |
| SIGLEC1 | -4.4583 | 2.21E-65 |
| EDNRB | -4.4973 | 8.47E-11 |
| VSIG4 | -4.5093 | 3.05E-34 |
| FCN1 | -4.5627 | 1.75E-90 |
| CPVL | -4.5690 | 5.43E-17 |
| HS3ST2 | -4.6059 | 5.77E-14 |
| RARRES1 | -4.6402 | 7.02E-40 |
| ADORA3 | -4.6911 | 2.16E-24 |
| ASGR2 | -4.8000 | 2.65E-26 |
| LEP | -4.9172 | 2.39E-10 |
| CXCL11 | -4.9181 | 1.02E-07 |
| OLFML2B | -5.0181 | 1.52E-48 |
| DEFA3 | -5.0279 | 3.52E-09 |
| GGTA1 | -5.0798 | 7.67E-10 |
| RPL7AP64 | -5.2258 | 2.16E-16 |
| LILRB5 | -5.3127 | 7.30E-21 |
| CACNA2D3 | -5.3522 | 0.000265827 |
| C1QB | -5.7529 | 1.46E-15 |
| TMEM37 | -6.0504 | 1.18E-16 |
| LOC101928228 | -6.3358 | 4.07E-14 |
| DEFA4 | -6.3559 | 1.54E-10 |
| C1QA | -6.4103 | 2.68E-23 |
| CRISP3 | -7.0401 | 1.73E-39 |
| C1QC | -7.1919 | 1.06E-49 |
| FOLR2 | -7.2586 | 4.82E-25 |
| CD163 | -7.2846 | 1.45E-55 |
| MS4A6A | -7.3661 | 1.94E-61 |
| CAMP | -7.9365 | 8.81E-23 |
| CD163L1 | -8.4418 | 3.29E-39 |
| F13A1 | -8.4620 | 2.90E-86 |
| RNASE1 | -8.5473 | 8.72E-33 |
